# Iterative Modeling via Structural Diffusion (IMSD): Exploring Fold‐Switching Pathways in Metamorphic Proteins Using AlphaFold2‐Based Generative Diffusion Model UFConf

**DOI:** 10.1002/prot.70050

**Published:** 2025-09-24

**Authors:** Dmitrii A. Luzik, Nikolai R. Skrynnikov

**Affiliations:** ^1^ Laboratory of Biomolecular NMR St. Petersburg State University St. Petersburg Russia; ^2^ Department of Chemistry Purdue University Indiana USA

**Keywords:** folding/unfolding intermediates, generative diffusion model, metamorphic proteins, modeling of fold‐switching pathways, parallel cascade selection molecular dynamics

## Abstract

Metamorphic proteins (MPs) can fold into two or more distinct spatial structures. Increasing interest in MPs has spurred the search for computational tools to predict proteins fold‐switching potential and model their refolding pathways. Here we address this problem by using the recently reported generative diffusion predictor UFConf, based on the AlphaFold2 network. We have developed a new UFConf‐driven algorithm dubbed IMSD (iterative modeling via structural diffusion) to model the MP's path from one conformational state to another. In brief, we begin with the experimental structure of state A, perturb it through the “noising” process, and infer a number of models (replicas) through the reverse diffusion or “denoising” process. From this set of models, we choose the one that is closest to the alternative structure B; then we use it as a starting point to perform another round of noising/denoising and thus generate the next batch of replicas. Repeating this process in an iterative fashion, we have been able to map the entire path from state A to state B for metamorphic proteins GA98, SA1 V90T, and the C‐terminal domain of RfaH. The obtained representation of the fold‐switching pathways in these MPs is consistent with the dual‐funnel energy landscape observed in the previous modeling studies and shows good agreement with the available experimental data. The new UFConf‐based IMSD protocol can be viewed as a part of the emerging generation of modeling tools aiming to model protein dynamics by means of deep learning technology.

## 1 Introduction

The sequence‐structure–function paradigm, suggesting that protein sequence uniquely defines its three‐dimensional structure, which in turn determines its function, has dominated the fields of biochemistry and structural biology for almost 70 years since the first protein structure has been solved [1]. This concept has been expanded with the discovery of intrinsically disordered proteins (IDPs), which fulfill their function despite the lack of a well‐defined 3D structure [2]. Another significant milestone was the discovery of metamorphic proteins (MPs), which can adopt two or more distinct spatial structures [3, 4]. In contrast to IDPs, which interconvert between many poorly ordered species, MPs typically switch between two well‐structured states that are both sufficiently stable and represent different protein folds.

An ideal example of a metamorphic protein represents a mixture of two structurally distinct protein forms that are both significantly populated, allowing for their direct experimental detection and structural characterization (e.g., by means of NMR spectroscopy). However, in practice, one of the states is often dominant while the other is only sparsely populated, which precludes its direct experimental observation. In this situation, investigators usually try to adjust experimental conditions, such as pH, temperature, or salt concentration, seeking to boost the population of the minor state [5]. Furthermore, point mutations are often introduced into the protein sequence or the sequence is otherwise altered (e.g., truncated) to drive the protein into its presumed minor state [6, 7]. These manipulations may result in a situation where the altered protein adopts a new fold, different from the original version. Strictly speaking, such systems should not be classified as metamorphic since the fold switching is caused by changes to protein sequence [8]. Nevertheless, one can argue that the alternative fold is, in fact, accessible to the original protein and point mutation (or another modification) simply helps to expose these species. In our paper, we accept this latter, broader interpretation of what constitutes a metamorphic protein.

Given the difficulties with experimental detection of sparsely populated alternative states, only relatively few such systems have been described to date. However, according to some estimates up to 4% of proteins in the Protein Data Bank (PDB) may be classified as fold‐switching [9]. Indeed, it has been shown that the fold‐switching phenomenon is likely a result of evolutionary selection, rather than just a random feature [10, 11]. The first discovered MP where metamorphosis is relevant to protein function is chemokine XCL1, which interconverts between chemokine‐like and all‐β dimeric fold [3, 10]. While the chemokine fold interacts with a G‐protein coupled receptor, the all‐β form binds glycosaminoglycans [12]. Other notable examples of proteins where metamorphism has proven functional significance are KaiB involved in cyanobacterial circadian rhymes [13], human spindle checkpoint protein Mad2 [14], and bacterial transcription factor RfaH [15].

Increasing interest in MPs has spurred the search for computational tools to model their refolding pathways. In theory, molecular dynamics (MD) simulations can be used to model fold‐switching transition with atomic‐level resolution. However, the time scale of this process is prohibitively long for conventional MD modeling. To address this issue, a number of enhanced sampling methods have been applied, such as replica exchange and modeling employing limited data [16, 17]. For bigger systems, MD simulations using coarse‐grained structure‐based models have also been implemented [18].

At the same time, various structure‐prediction tools have been employed to appraise proteins' fold‐switching potential. AlphaFold2 (AF2) [19], a revolutionary machine learning tool for prediction of protein structures, showed little success in predicting alternative states of metamorphic proteins [20]. Recently, a number of modifications to the AF2 training protocol have been proposed with the explicit purpose to model proteins' conformational variability, including alternate folds in metamorphic proteins [21, 22]. While the initial results appear promising, the ability of the AF2‐based approaches to predict fold‐switching behavior apparently remains limited [23].

A very recent addition to the field of protein structure prediction is generative diffusion models (GDMs) [24, 25, 26, 27]. These algorithms build protein structures from individual amino acids through a process that can be described as diffusion in a potential, where the potential is generated by a suitably trained neural network. To train GDMs, protein coordinates are first corrupted via the so‐called forward diffusion (also described as “noising”); the network then learns to eliminate noise and recover the original structure through the so‐called reverse diffusion (“denoising”). For example, the popular generative model RFdiffusion is based on the previously developed neural network RoseTTAFold [28]; this model has shown excellent performance in protein structure predictions, as well as protein binder design, enzyme active site scaffolding, and so forth [24].

In this work, we make use of the recently reported generative diffusion algorithm UFConf [29], which is built around the suitably modified AlphaFold2 network. Modeling by UFConf starts with a protein structure, which is subjected to a forward diffusion (noising) run. In brief, the protein is broken down into individual amino acids, which are then diffused for a period of time t via translational and rotational diffusion. Parameter t is confined to the interval $\left[0,1\right]$, where the value of 0 corresponds to no noising and the value of 1 corresponds to (nearly) completely randomized placement of the protein's constituent amino acids. Starting with this fragmented model, UFConf algorithm regenerates protein structure via the reverse diffusion (denoising) process guided by the learned probability distribution. The authors have demonstrated that UFConf can successfully predict various conformational rearrangements in proteins such as membrane transporter, multidomain filament protein and a kinase [29].

First, we asked ourselves if UFConf can successfully capture both protein folds when starting from a fully randomized initial configuration (i.e., without any specific structural input, but rather based on a sequence alone). The tests have been conducted on six metamorphic proteins: GA98, SA1, full‐length RfaH, C‐terminal domain of RfaH, XCL1, and Mad2. For five out of six, we found that UFConf successfully approximates one of the folds, but not the other one. In only one case, that of full‐length RfaH, reasonable approximations have been obtained for both folds. At the same time, we also observed that UFConf generates multiple species that resemble intermediates on the path from one fold to another. We reasoned that UFConf, which is based on AlphaFold2, possesses some general knowledge of the principles of protein architecture. Consequently, it can recreate not only the stable folds but also produce realistic models for unfolding/folding intermediates.

Motivated with this idea, we have developed the new IMSD (iterative modeling via structural diffusion) protocol to trace the path of a metamorphic protein from one fold to another. In brief, we begin with the experimental structure of state A, perturb this structure through the noising process, and then infer a new structural model through the reverse diffusion process. By repeating this procedure multiple times, we generate a batch of models (replicas), from which we choose the one that is closest to the alternative state B. We then use this particular model (termed iteration minimum, or IM) as a starting point, perform “noising–denoising” routine, and thus generate the next batch of models, from which we again select the one that is closest to state B, etc. By iterating this process, we have been able to map the entire path from state A to state B for several metamorphic proteins.

Clearly, the IMSD protocol represents a steered algorithm to model the transition path from fold A to fold B (the reverse transition from B to A can be modeled as well). Note that the steering is performed in a fairly gentle manner—through selection of the IM states, which are used to iterate the process of UFConf model‐building. It does not interfere in any way with the UFConf procedure per se, and therefore we argue that UFConf retains its ability to build meaningful models, which can approximate different protein states (including folding/unfolding intermediates). The output of the IMSD procedure is the series of the IM states on the path from A to B. As it turns out, these IM states can be interpreted as fold‐switching intermediates or, more generally, on‐path species, as supported by the experimental evidence.

Generally, we believe that machine learning engines, such as AF2‐based tool UFConf, have a fundamental ability to model protein folding, unfolding, or refolding. In this paper, we present the IMSD scheme, which employs UFConf to model the process of fold‐switching by metamorphic proteins. The new scheme is sufficiently robust, offering an attractive alternative to more computationally intensive methods using enhanced sampling MD techniques.

## 2 Results and Discussion

### 2.1 Can UFConf Predict Two Metamorphic Folds in a Single Run?

As discussed above, UFConf is a diffusion‐based generative model, which aims to sample protein conformations using both protein sequence and structure as an input [29]. First, the program (partially) disassembles the structure, then reassembles it by means of the diffusion algorithm driven by a suitably modified AlphaFold2 network. In our simulations, one UFConf run produces 100 replicas, representing a range of structures consistent with the input. In the case of metamorphic proteins, it is conceivable that UFConf can recover both characteristic folds among the 100 output conformers. To test this conjecture, we have conducted the tests using six well‐characterized metamorphic systems.

For the sake of convenience, we label the two states of metamorphic protein “ground state” (GS) and “alternative state” (AS). In doing so, we assume that the ground state is the one that is dominant under normal conditions (room temperature, neutral pH, close to physiological salt concentration, absence of the bound ligand, etc.). While the choice between GS and AS designations may not always be straightforward, in most cases it is sufficiently clear‐cut.

To test the ability of UFConf to recover both metamorphic folds in a single run, we set the diffusion‐time parameter to t = 1.0. With this setting the program generates an initial spatial distribution of amino acids which is completely random. Thus, any prior structural information is erased and the input is limited to protein sequence alone. In this manner, we avoid biasing UFConf toward the starting structure, hoping to recover both GS and AS folds in a single run.

As a first example, we discuss the well‐known metamorphic protein GA98 [6]. This system is a product of a rational design approach. As a starting point, Bryan and co‐workers used two different domains from the streptococcal protein G [6, 30]. The native GA domain consists of 45 structured residues, adopts 3α fold and, characteristically, binds to human serum albumin (HSA). The native GB domain consists of 56 structured residues, adopts 4β + α fold and, characteristically, binds to the constant fragment (Fc) of immunoglobulin G (IgG). The two domains, GA and GB, have no significant sequence similarity. In their work, Bryan et al. have redesigned both GA and GB, seeking to bring their sequences closer to each other. These efforts resulted in a pair of constructs, GA98 and GB98, which are 98% sequence identical (differing only by a single substitution, L45Y). Despite this very high level of homology, GA98 retains its 3α fold and binding propensity to albumin, while GB98 retains its 4β + α fold and binding propensity to IgG.

At the same time, it has been found that GA98 has affinity for IgG. This led the authors to suggest that GA98 is, in fact, a metamorphic protein, which alternates between the dominant 3α species (ground state, identified by protein's binding to albumin) and low‐populated 4β + α species (alternative state, identified by protein's binding to IgG) [6]. Later, it has been inferred that GB98 is also a metamorphic protein, where the ground state and the alternative state are reversed compared to GA98 [31, 32]. The structures of both GA98 and GB98 were subsequently solved by means of solution NMR spectroscopy [33], paving the way to simulation studies of fold‐switching in this system [34, 35].

**FIGURE 1:**
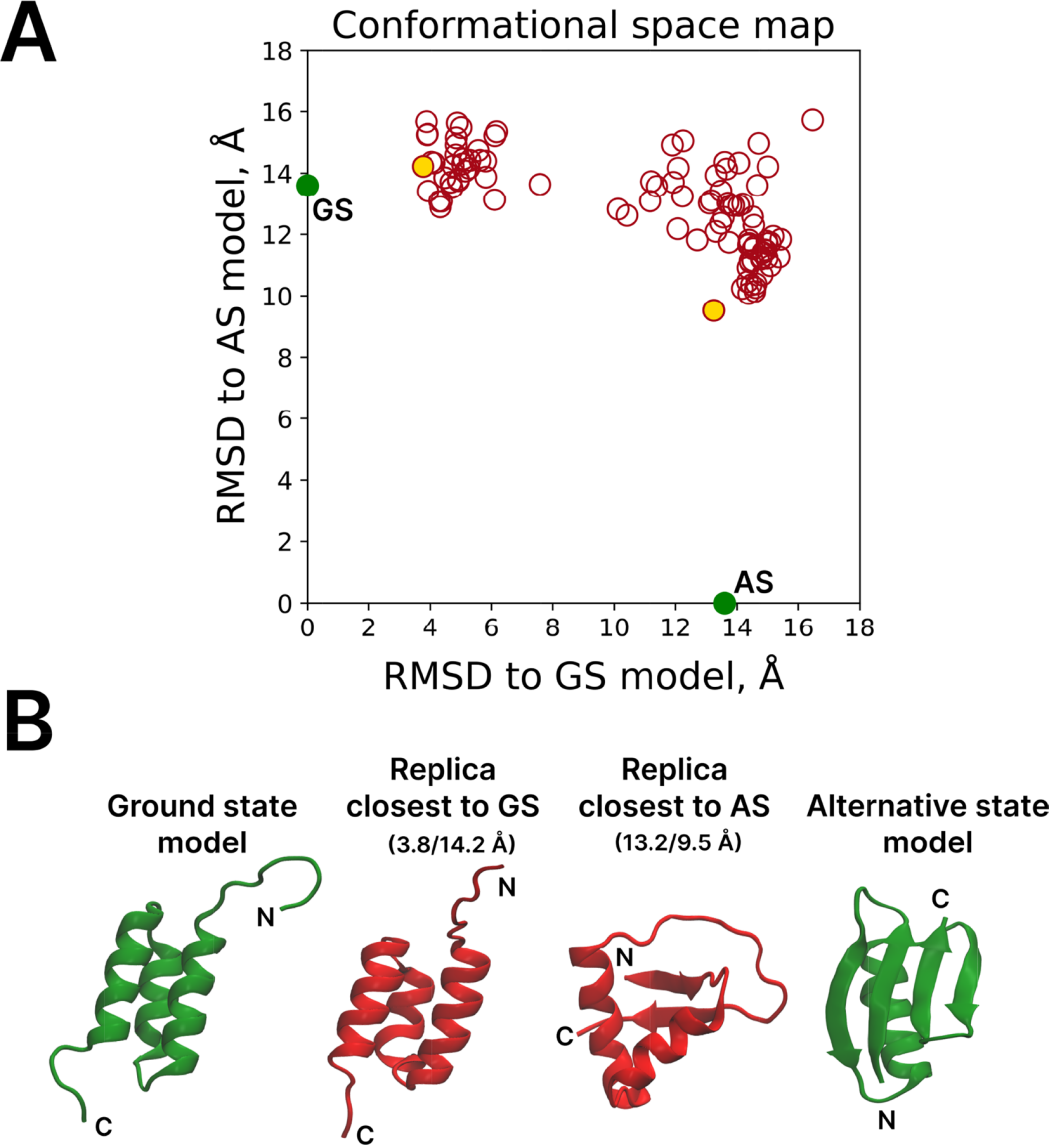
Single‐run UFConf predictions for metamorphic protein GA98 (t=1, 100 replicas). (A) Conformational space map where each replica is mapped according to its backbone *rmsd* relative to the pair of reference structures, GS (first model from the NMR‐determined structure 2LHC) and AS (first model from the NMR‐determined structure 2LHD). Gold‐filled circles represent the replicas with the lowest *rmsd* to the GS and AS models. (B) Cartoon representation of GS and AS models (green), as well as UFConf replicas with the lowest *rmsd* to the GS and AS models (red). For the UFConf replicas, the values of *rmsd* to the GS and AS models are indicated in brackets above the respective models. In addition, we also conducted a UFConf test that generated 1000 replicas; the results proved to be similar to those illustrated in this figure.

For this system, we executed the UFConf run using GA98 sequence (t = 1.0) and thus generated 100 predicted structures (replicas) of the protein. For each replica, we calculated the root‐mean‐square deviation (*rmsd*) of the backbone atomic coordinates relative to the pair of reference structures, GS (3α fold, PDB id 2LHC) and AS (4β + α fold, PDB id 2LHD). The results are mapped in Figure 1A, where every circle represents a single replica. Those replicas that are closest to the GS state and AS state are indicated by gold‐filled circles. They are also shown in Figure 1B (red models) along with the reference structures (green models).

The results in Figure 1 demonstrate that UFConf has successfully reproduced the GS fold of GA98 (cf. two models on the left of Figure 1B), but failed to recover the AS fold. Nonetheless, it generated a number of replicas that are closer to AS rather than GS (i.e., fall under the diagonal in the map Figure 1A). Characteristically, these replicas feature the nascent β‐sheet structure as found in the AS fold (cf. two models on the right of Figure 1B).

The question is how to interpret those UFConf replicas, which are only remotely similar to both GS and AS. Given the UFConf mastery of protein architecture, we argue that these are not simply failed models, but rather feasible structural states on the path from GS to AS (some of them are probably sufficiently stable to be viewed as fold‐switching intermediates). This idea led us to formulate the iterative IMSD protocol, detailed in the next section.

In addition to GA98, we have also tested UFConf using a complementary GB98 sequence. The latter sequence differs by only one mutation, but this single mutation leads to a reversal of metamorphic states: the 4β + α fold becomes dominant (GS), while 3α fold becomes rare (AS). The results of the UFConf run performed on GB98 are illustrated in Figure S1. In this case, UFConf produced one replica that recapitulates the GS fold, as well as some replicas that show loose similarity to the AS fold. All other replicas fall somewhere in between GS and AS, consistent with their interpretation as transition species.

We have also conducted analogous UFConf tests on other metamorphic proteins, see Figure S2. For SA1, XCL1, Mad2, and the isolated C‐terminal domain of RfaH, the UFConf‐generated replicas gravitate toward the GS fold, while showing a range of species that deviate to various degrees from the GS model. One special case is full‐length RfaH, which has been recovered in an open conformation with its C‐terminal domain adopting 5β fold (corresponds to a low‐populated AS state). At the same time, UFConf also generated a number of closed conformations of RfaH, where the C‐terminal domain is transformed into an α‐helical hairpin (resembling the dominant GS state). More information about this system can be found in Section 2.4.

We conclude that, with some exceptions, UFConf usually recovers the ground state of the metamorphic protein and fails to predict its alternative state. In this sense, UFConf is similar to AlphaFold2, on which it relies to perform the reverse‐diffusion step [36]. However, we note that UFConf generates a multitude of conformational species on the path from GS to AS, which can be interpreted as fold‐switching intermediates. Experimental evidence in support of this hypothesis will be discussed in what follows.

### 2.2 IMSD Modeling of Fold‐Switching Transition in GA98

In this section, we model the fold‐switching transition in GA98 using the UFConf‐based IMSD (iterative modeling via structural diffusion) scheme. The details of this protocol are described below.

Same as before, we rely on two reference structures, representing the protein's ground state (3α fold, PDB deposition 2LHC, leftmost model in Figure 1B) and alternative state (4β + α fold, PDB deposition 2LHD, rightmost model in Figure 1B). We begin the IMSD simulation with the UFConf run which partially disassembles the GS structure (t = 0.5) and uses the resulting variable arrangements of amino acids as starting points to build 100 new structural models (replicas). Since the initial structure is only partially randomized, all replicas turn out to be rather similar to the GS.

From the so generated pool of replicas, we select the one with the lowest *rmsd* to the AS model; this replica is called the Iteration Minimum (IM) state. The IM model is then used as a starting structure to launch another UFConf run and identify the next IM, and so the process is iterated for a number of times. As the simulation progresses, the IM states gradually become less similar to the GS and more similar to the AS, thus modeling the fold‐switching transition.

At some point the latest in the series of IM states approaches to within several angstroms of the (target) AS state. At this stage we want to reduce the perturbing effect on the system, that is, reduce the parameter t, so that the next UFConf search is localized in the vicinity of AS. Since t controls the extent of noising (i.e., loss of structure) it is convenient to think of this parameter as effective temperature. Early in the IMSD simulation we are interested in heating the system so that it can climb out of the “energy well” associated with GS, but late in the simulation we want to cool the system down such that it can settle in another “energy well” representing the AS.

To implement this agenda, we adopted the following scheme. During the course of the simulation, when *rmsd* between the current IM and AS falls below 5 Å, we reduce t from 0.5 to 0.4; when *rmsd* falls further to less than 3 Å, we lower t to 0.3. Finally, when *rmsd* reaches 2 Å, we assume that at this level of accuracy IM provides a *bona fide* model of the AS fold and thus terminate the simulation. In what follows, we differentiate between the main stage of the IMSD simulation (t = 0.5) and the refinement stage (t = 0.4 and 0.3).

The choice of parameters in the above scheme is clearly empirical, but it works well for two of the metamorphic proteins at hand. One can envision that a more sophisticated adaptive algorithm may be developed in the future, where t is automatically adjusted in response to the current progress of the simulation on the path from GS to AS. However, in this report we merely seek to demonstrate the principle of IMSD modeling and, therefore, opt for a simple empirical protocol.

**FIGURE 2:**
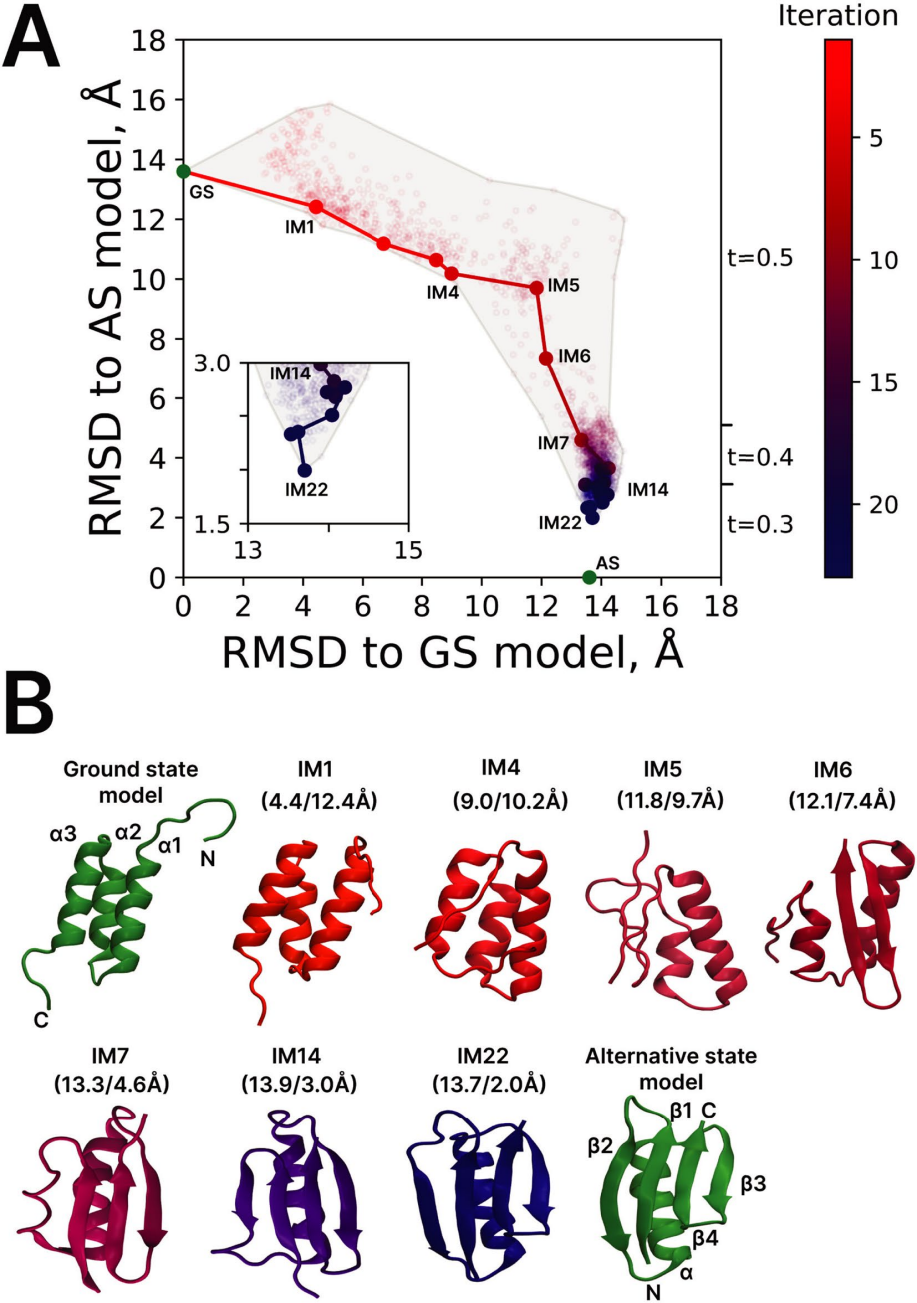
IMSD simulation of fold‐switching transition in GA98. (**A**) Conformational space map where each structural model is mapped according to its backbone *rmsd* relative to the GS and AS models. Bright solid circles represent the IM models, pale empty circles represent all other UFConf‐generated replicas (colored from red to blue according to iteration number, cf. color bar on the right); green circles represent the GS state (starting model) and the AS state (target). The gray shaded region is drawn around all replicas generated during the simulation. Shown in the insert is the magnified image of the final portion of the IMSD trajectory (iterations 14 to 22). The ranges of parameter t used for UFConf modeling are indicated on the right side of the plot. (B) Cartoon representation of GS and AS models (green), as well as selected IM models (colored from red to blue), representing the key transformations on the path from GS to AS. The *rmsd* values relative to GS and AS models are indicated in brackets above each IM model. The secondary‐structure elements in the reference structures and their constituent residues are as follows: GS—α1 (9–22), α2 (27–35), α3 (39–51), AS—hairpin β1–β2 (2–19), α (23–37), hairpin β3–β4 (42–55). The evolution of the secondary structure throughout the trajectory is visualized in Figure S3A.

The IMSD simulation of the GA98 transition from 3α fold to 4β + α fold is illustrated in Figure 2. Same as before, the 2D map characterizes each UFConf‐generated replica in terms of backbone *rmsd* relative to the GS and AS states; the IM conformers are indicated by solid circles with all other replicas shown as pale empty circles. The progression of the simulation from the first to the last iteration is coded by color (changing from red to blue according to the color bar scale). The initial GS state (green circle) and the consecutive IM states are connected by straight line segments, forming a trajectory on the map. The entire area containing the UFConf‐generated replicas is enclosed in a gray contour. Shown on the right side of the map are the ranges indicating where one or the other t value has been used.

Certain representative IM states are labeled in the map according to their iteration number. These states are also visualized in Figure 2B (with colors ranging from red to blue), along with the GS and AS states (green). Note that in terms of their physical relevance, IMs are not different from other UFConf‐generated replicas, that is, the term “iteration minimum” does not imply that these conformers are energetically favored over others.

Surveying the trajectory in Figure 2A, we observe that the simulation that starts from the GS model eventually arrives at the state within 2 Å of the AS model. In other words, during the course of the simulation, the protein has switched folds (the feat that proved to be impossible in a single UFConf run, see Section 2.1). The trajectory consists of two branches. First, the system moves in a general direction from left to right (from GS to IM5), with *rmsd* to GS progressively increasing but relatively little change in *rmsd* to AS. This branch corresponds to partial unfolding of the GS structure. After that, the trajectory makes a turn and starts moving downward (from IM5 to IM22), with *rmsd* to GS showing little change but *rmsd* to AS progressively decreasing. This branch describes the formation of the AS fold.

The gray shaded region in Figure 2A that is drawn around the entire grouping of replicas on the map has the characteristic boomerang shape, matching the above description of the transition path. This distinctive shape has also been found in the enhanced MD simulations of fold‐switching transitions in a two‐funnel energy landscape [37, 38]. Note how the AS funnel narrows toward the bottom, see Figure 2A. This is undoubtedly a physical characteristic of the folding funnel, which is reproduced in the IMSD simulation. In part, this effect is due to the lowered t value at the refinement stage, beginning from IM8, but it is already noticeable during the main stage, from IM5 to IM7.

Let us now trace the main events on the path from the GS to AS state, as illustrated in Figure 2B. The state IM1 is rather similar to its parent GS model. The all‐helical fold is preserved for another three iterations, but in IM4 the separation grows between α3 and the other two helices, and α3 also frays at the C‐terminus. The next iteration minimum, IM5, shows the most significant loss of structure, with α3 fully dissolved and α2 shortened. Starting from this point, the alternative fold begins to form. Already in IM6, the disordered C‐terminal portion of the peptide chain folds into β3–β4 hairpin. At the same time, α1 shortens while α2 expands (note the change in perspective in plotting IM6). The latter helix corresponds to helix α in the AS state; it remains stable until the end of the simulation.

In the next state, IM7, we observe the nucleation of the β1–β2 hairpin structure at the N‐terminus of the polypeptide chain. However, the strand β1 is only partially formed at this stage, and some distorted helical geometry inherited from α1 is observed in the region 10–18. A more convincing version of β1–β2 is found in IM14 and several subsequent states. Finally, in IM22 we observe the fully formed β‐sheet, which is similar to the one observed in the AS state.

At this point it is appropriate to ask whether the results of the IMSD simulations, such as illustrated in Figure 2, are reproducible. To address this question, we have recorded a duplicate trajectory, see Figure S4. While some of the details differ, the duplicate trajectory features the same key events occurring in the same order: dissolution of α3, followed by formation of β3‐β4 and extension of α2 (turning into α), then emergence of β1 and, finally, transformation of the remaining portion of α1 into the edge strand β2.

In addition, we have also recorded a trajectory simulating the reverse transition from AS to GS, see Figure S5. In this latter trajectory we observe the same key transformations, but in the reverse order. Already after the first iteration, the AS structure undergoes a major change, with β1–β2 hairpin replaced by the nascent α1 helix. This happens rapidly because the AS state is unstable and readily unfolds. Then after several more iterations β3–β4 hairpin disappears giving way to α3. During the refinement stage the three helices, α1, α2, and α3, are packed against each other until finally the simulation arrives at the model within 2 Å of the GS state.

How does all of this correlate with the available experimental data, as well as bioinformatics data? The early loss of α3 during the course of the GS to AS transition is not surprising. Indeed, secondary structure predictors AGADIR [39], Jpred4 [40], and PSSPred [41] all indicate that this region has low helicity, but sizeable β‐sheet propensity. Furthermore, mutation‐based Φ analysis [42] of GA88 suggests that the all‐helical fold of this protein is underpinned by the cluster of residues in helices α1 and α2 [43]. Almost all of these key residues are found in the surviving helical core of IM5 (which is our most disordered IM state). This core also includes residue L20, which is the site where a single mutation can cause fold‐switching [33].

A significant amount of experimental data has been gathered on the formation of 4β + α fold in GB1 protein (which is 62% sequence‐identical to GA98). In particular, Blanco et al. used solution NOE measurements to demonstrate that the peptide corresponding to β3–β4 sequence has a strong propensity to form β‐hairpin, above 40%, whereas the peptide corresponding to β1–β2 sequence has no such propensity [44]. Kuszewski et al. measured H/D exchange protection factors, concluding that the folding nucleus of GB1 involves β3–β4 hairpin that is packed against helix α. At the same time, the edge strand β2 is the last addition to the β‐sheet and is not a part of the folding nucleus [45]. These conclusions were later confirmed by the mutation study by McCallister and co‐workers [46]. The so‐identified folding nucleus includes residues T25 and L45, where point mutations can trigger fold switching [33]; it also contains most of the key conserved residues that control the fold [47]. These experimental observations are in agreement with our IMSD simulations, where we observe early formation of the β3–β4 hairpin and its packing against the helix α (IM6), whereas β2 is not fully established until the end of the simulation.

Thus we conclude that our IMSD trajectory recapitulates the main features of the unfolding/folding processes as observed in this system. These observations have also found support in a number of prior modeling studies [48, 49, 50, 51].

The archetypal metamorphic protein GA98 is also well suited for negative‐control simulations. To this end, we set up a simulation where the AS model has been replaced with a 56‐residue decoy representing the N‐terminal SH3 domain from adapter protein Grb2 (PDB id 1AZE [52], chain A). The topology of the decoy is entirely different from that of the actual alternative state (the sequence is also different, but this is inconsequential since only the backbone *rmsd* to AS is evaluated in our protocol). In recording this negative‐control trajectory, we can anticipate two outcomes. If the simulation succeeds in refolding GA98 into a non‐native SH3 fold, that would clearly constitute a failure of the IMSD methodology. Conversely, if the simulation leads to partial unfolding of GA98 but shows no evidence of refolding, that would be consistent with expectations and confirm the validity of the method.

The results of this negative‐control simulation are summarized in Figure S6. The structure of GA98 has indeed undergone some changes consistent with partial unfolding. However, the resulting IM states show no similarity whatsoever with the decoy fold. Therefore, this test confirms the sound nature of the IMSD approach. In addition, we also conducted a similar test on non‐metamorphic protein ubiquitin paired with the N‐terminal domain of calmodulin (PDB ids 1UBQ and 1F70, respectively). As one may expect, highly stable ubiquitin has undergone little conformational change and showed no signs of fold switching, see Figure S7.

### 2.3 IMSD Modeling of Fold‐Switching Transition in SA1 V90T

SA1 is a recently reported artificial 95‐residue MP based on S6 ribosomal protein from *T. thermophilus* [53]. Briefly, residues 11 to 66 of the (slightly altered) S6 parent protein have been replaced by the 56‐residue‐long sequence of the GA domain (see Section 2.1). The so‐obtained chimeric construct has a potential ability to adopt two different folds—either α/β‐plait fold, which is native to the S6 ribosomal protein, or otherwise all‐helical fold encoded in the GA sequence. This construct was further evolved *in silico* with the goal to accommodate the GA‐type sequence within the native S6 fold. The design process led to SA1, which has S6‐like α/β fold but apparently can convert to the alternative all‐helical state [54].

Metamorphic nature of SA1 has been put on display in the follow‐up study [55]. As it turns out, a single mutation in the SA1 sequence, V90T, unmasks the presence of the alternative state, making it possible to observe the mixture of GS and AS species in an HSQC spectrum. Hence, SA1 V90T represents a clear‐cut case of protein metamorphism. Interestingly, at 30°C this mutant mostly populates the α/β state, while at 5°C the equilibrium shifts toward an all‐α conformation. As it appears, the relative stability of α/β decreases at lower temperatures, reminiscent of cold denaturation [55].

**FIGURE 3:**
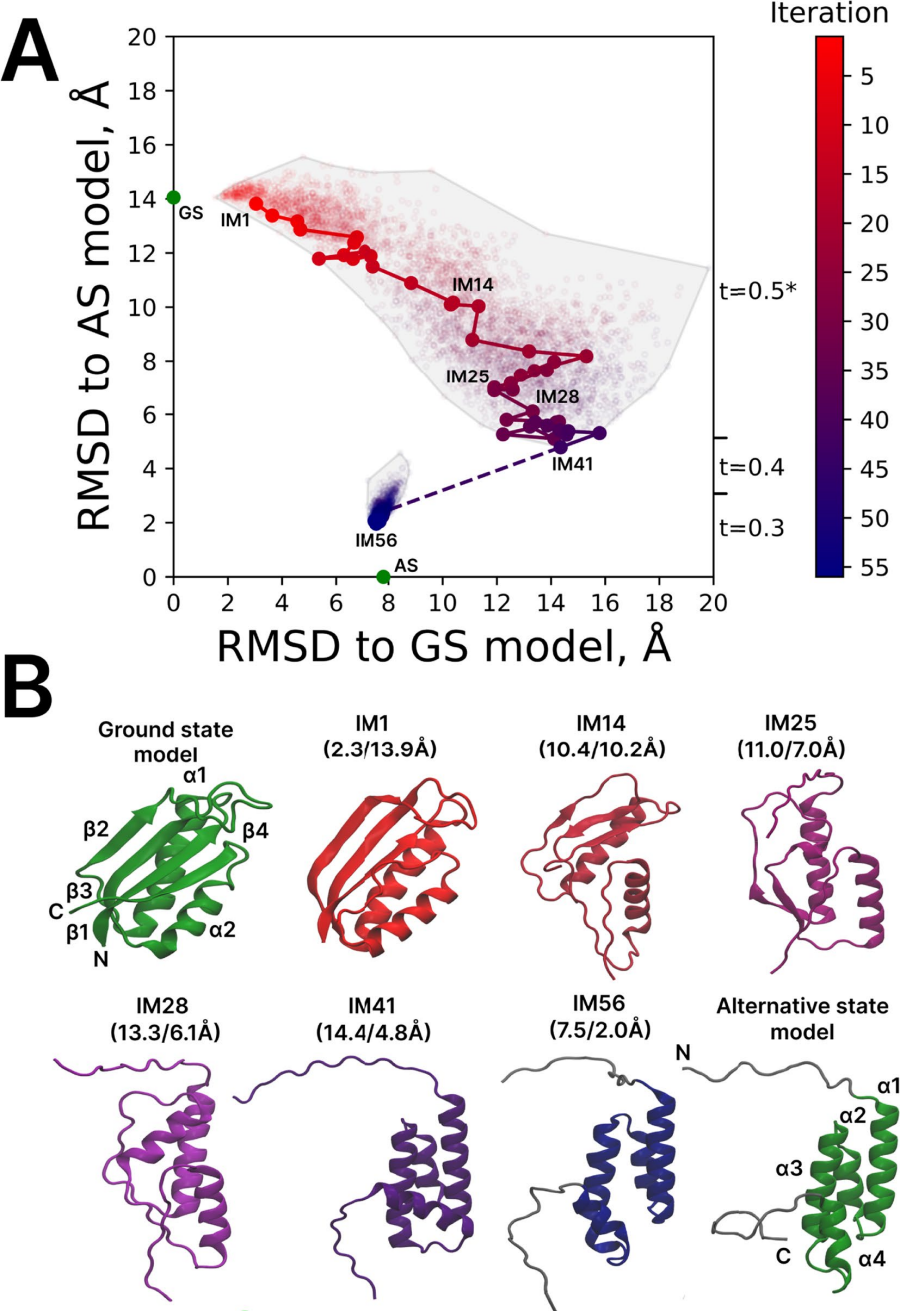
IMSD simulation of fold‐switching transition in SA1 V90T. (A) Conformational space map where each structural model is mapped according to its backbone *rmsd* relative to the GS and AS models. The ranges of parameter t used for UFConf modeling are indicated on the right side of the plot (*except the first iteration where t = 1.0). Dashed line indicates the point where the rule to calculate *rmsd* is changed (beginning from this point only residues 15–80 are included in the calculations). The AS state is mapped using this updated definition of *rmsd*. (B) Cartoon representation of GS and AS models (green), as well as selected IM models (colored from red to blue), representing the key transformations on the path from GS to AS. The secondary‐structure elements in the reference structures and their constituent residues are as follows: GS—β1 (1–10), α1 (16–33), β2 (40–43), β3 (59–67), α2 (69–79), β4 (85–92), AS—α1 (16–31), α2 (37–44), α3 (49–63), α4 (71–79). The evolution of the secondary structure throughout the trajectory is visualized in Figure S3B. Flexible N‐ and C‐terminal tails in IM56 and AS (residues 1–14 and 81–95) are painted gray; these segments are not included in the calculations of *rmsd* beginning from the 42‐nd iteration.

Here we set out to model the fold‐switching transition in SA1 V90T using the newly developed UFConf‐based IMSD protocol. The GS structure of SA1 V90T has been solved at 30°C by solution‐state NMR using chemical shift data as well as NOE restraints (α/β fold, PDB id 8E6Y). In contrast, the AS structure has been determined at 5°C using backbone chemical shifts alone; the corresponding structural model has been generated using the program CS‐Rosetta [56]. Although this model is likely sufficiently accurate, it is considered to be less reliable than a *bona fide* NMR structure and, therefore, has been deposited to a special PDB‐IHM database [57] (4α fold, PDB‐IHM id 9A20). The pair of reference structures, representing the GS and AS species of SA1 V90T, are shown in Figure 3B (green models); recall that the GS/AS nomenclature in this paper reflects the balance of populations at or near the ambient conditions.

Before we turn to the discussion of the IMSD simulation, we note that the recent structure 8E6Y, as well as SA1 structure 7MN1 and PDB‐IHM model 9A20, are all outside of the AlphaFold2 training set and therefore are unfamiliar to UFConf. However, a number of S6‐like proteins that are homologous to SA1 and possess α/β fold have been a part of the said training set (e.g., the native S6 ribosomal protein that has 57% sequence similarity to SA1 V90T). As is the case with nearly all metamorphic proteins, a single UFConf run consistently predicts the GS structure for SA1 V90T and fails to identify the alternative state; see Figure S2.

The IMSD protocol to simulate fold‐switching transition in SA1 V90T required some modifications. Our initial attempts to conduct the simulation beginning with t = 0.5 were unsuccessful. After 50 iterations, such IMSD trajectories produced only GS‐like structures with minor variations (not shown). To overcome this problem and drive the protein out of its “GS well,” we chose to (effectively) heat the system by using t = 1.0 in the first iteration. After this modification, the simulation has successfully run its course, generating a series of IM species on the path from GS to AS.

Besides, we note that there is some disparity between the GS model, which is fully structured, and the AS model, which features long disordered tails (as confirmed by spin relaxation measurements [55]). It obviously makes little sense to try to reproduce the conformation of these flexible tails while approximating the AS state. Therefore, we amended the operational definition of *rmsd* used in our protocol. Specifically, during the refinement stage, the calculation of *rmsd* was restricted to the structured portion of the AS state, residues 15 to 80. In this manner, the convergence of the IMSD simulation in the vicinity of the AS has been significantly improved.

The results from this amended simulation scheme are summarized in Figure 3. Note that the format of this graph is slightly different from Figure 2. The initial structure (GS, green circle in the map) is no longer connected to IM1. This is because the setting t = 1.0 has been used in the starting iteration, meaning that all structural information associated with the GS model is erased and, therefore, there is no structure‐based connection between GS model and IM1 model. Note also the dashed line between IM41 and the subsequent iteration minimum. This is the point where the simulation enters the refinement stage and the definition of *rmsd* is updated; as can be appreciated from the graph, the new *rmsd* values (calculated for residues 15–80) are substantially lower than the previous ones (calculated for residues 1–95).

At this point, we turn to the discussion of the IMSD simulation of SA1 V90T as illustrated in Figure 3. In this specific implementation of the protocol which initially employs t = 1.0, UFConf builds a number of structural models based on sequence information alone. All of these models are similar to GS, but some differences begin to emerge already at this stage. In particular, β4 strand at the edge of the β‐sheet is lost already in IM1.

Gradual deterioration of the remaining β‐sheet is observed in the next 20 or so iterations. For example, in IM14 we observe a residual β‐sheet structure comprised of a small portion of the edge strand β2, the opportunistic central strand (shifted relative to the original β3) and a portion of β1. At the same time, both α1 and α2 survive, with α2 now orthogonal to α1/β‐sheet and packed against them from the side.

Soon thereafter, the β structure is completely lost, but later it is briefly reestablished in IM24–IM27. In these iterations, we observe a pair of short strands, corresponding to the (shifted) β2 and β3. However, already in IM27, a new α‐helix begins to form in place of β3 (according to the AS nomenclature, this is α3). After that, the β sheet disappears for good, giving way to a fully helical arrangement, IM28 (see Figure 3B).

The last alternative‐state helix, α2 (AS nomenclature), begins to form in IM33. Eventually, in IM41 the three‐helical bundle α1–α2–α3 is formed with correct topology, in agreement with the AS model. However, the position of α4 is off—it is tilted relative to the other helices and packed against both α1 and α3. Finally, after 15 additional rounds of refinement (using the updated definition of *rmsd*) the system arrives at IM56, which closely resembles the AS fold. In this final conformation, α4 is packed against α3, the same as in the AS model; yet α4 remains tilted, in contrast to the AS model, where it is strictly parallel to α3 and the other helices. Considering this aspect, it may be hypothesized that IM56 is actually a better representation of the alternative state than the (chemical‐shift‐based) AS model.

At this stage, it would be appropriate to compare the predictions from the IMSD simulation to experimental data on unfolding/refolding intermediates of SA1 V90T. However, to the best of our knowledge, there are currently no experimental data that characterize the fold‐switching transition in this recently reported metamorphic protein. While relevant data can be found for the parent S6 ribosomal protein [58, 59, 60], the level of similarity between S6 and SA1 is insufficient to draw any meaningful conclusions. We therefore conclude that the IMSD model of the fold‐switching transition in SA1 V90T can be viewed as an *in silico* prediction awaiting future experimental verification.

### 2.4 IMSD Modeling of Fold‐Switching Transition in Isolated RfaH‐CTD

Both GA98 and SA1 V90T are designed MPs containing GA‐like sequences, which steer them toward an all‐helical form. In this section, we discuss a different example involving an isolated C‐terminal domain of RfaH.

Bacterial transcription antitermination protein RfaH consists of two domains, RfaH‐NTD and RfaH‐CTD. In its free state, this protein adopts an (autoinhibited) closed conformation where CTD is packed against NTD in the form of αα‐hairpin, see Figure S8A. After RfaH binds to *ops*‐paused RNA polymerase, the CTD dissociates from NTD and promptly refolds into β‐barrel, see Figure S8B. The so transformed CTD then engages the ribosome, thus initiating on‐site translation of the newly synthesized RNA. Upon termination of transcription, RfaH is released and reverts to its closed conformation [61].

While the metamorphic nature of RfaH has been recognized early on, no direct evidence of an alternative state (open conformation involving β‐barrel C‐terminal domain) can be found in the HSQC spectrum of the full‐length protein [7]. The fold switching can be detected, though, after binding to the *ops*‐paused transcription elongation complex [61]. Alternatively, the E48S mutation weakens the interdomain interaction, leading to a mixture of species (with differently folded CTD domains) observable in the HSQC spectrum [7]. This is the signature manifestation of a metamorphic protein.

The separation of the two domains by means of proteolytic cleavage results in the conversion of the CTD to the β‐barrel form. Likewise, when expressed as a separate construct, RfaH‐CTD adopts the β‐barrel conformation [62]. It can be further argued that both folds, β‐barrel and αα‐hairpin, are encoded in the RfaH‐CTD sequence and interdomain interaction simply favors the latter over the former. Hence, RfaH‐CTD can be regarded as a metamorphic protein in its own right [38]. Note that in isolation αα‐hairpins are normally unstable; they are stabilized by packing with other structural elements (e.g., an additional helix in a helical bundle) or by disulfide bridges [63, 64, 65]. Therefore, it is to be expected that αα‐hairpin form of RfaH‐CTD is a low‐populated alternative state.

In what follows, we use our IMSD scheme to investigate the fold‐switching behavior of an isolated RfaH‐CTD. As a ground‐state model we use the NMR structure of an isolated RfaH‐CTD (β‐barrel, PDB id 2LCL). In defining this model, we exclude the disordered N‐terminal tail and retain only the core portion of the domain, residues 115 to 162 (see Methods for additional comments). As for the alternative‐state model, there is no structural data for the presumed αα‐hairpin conformation of an isolated RfaH‐CTD. Therefore, we derive the model from the x‐ray structure of the full‐length RfaH in the closed conformation (PDB id 2OUG). The distinctive αα‐hairpin in this structure which corresponds to the CTD (painted green in Figure S8) is stabilized by the interaction with NTD. The crystallographically resolved portion of CTD is limited to residues 115 to 156 (segments 108–114 and 157–162, which are structured in the GS model, are apparently disordered in the AS state). Therefore, in the following IMSD simulations all *rmsd* values are calculated for this core portion of the sequence, residues 115 to 156.

**FIGURE 4:**
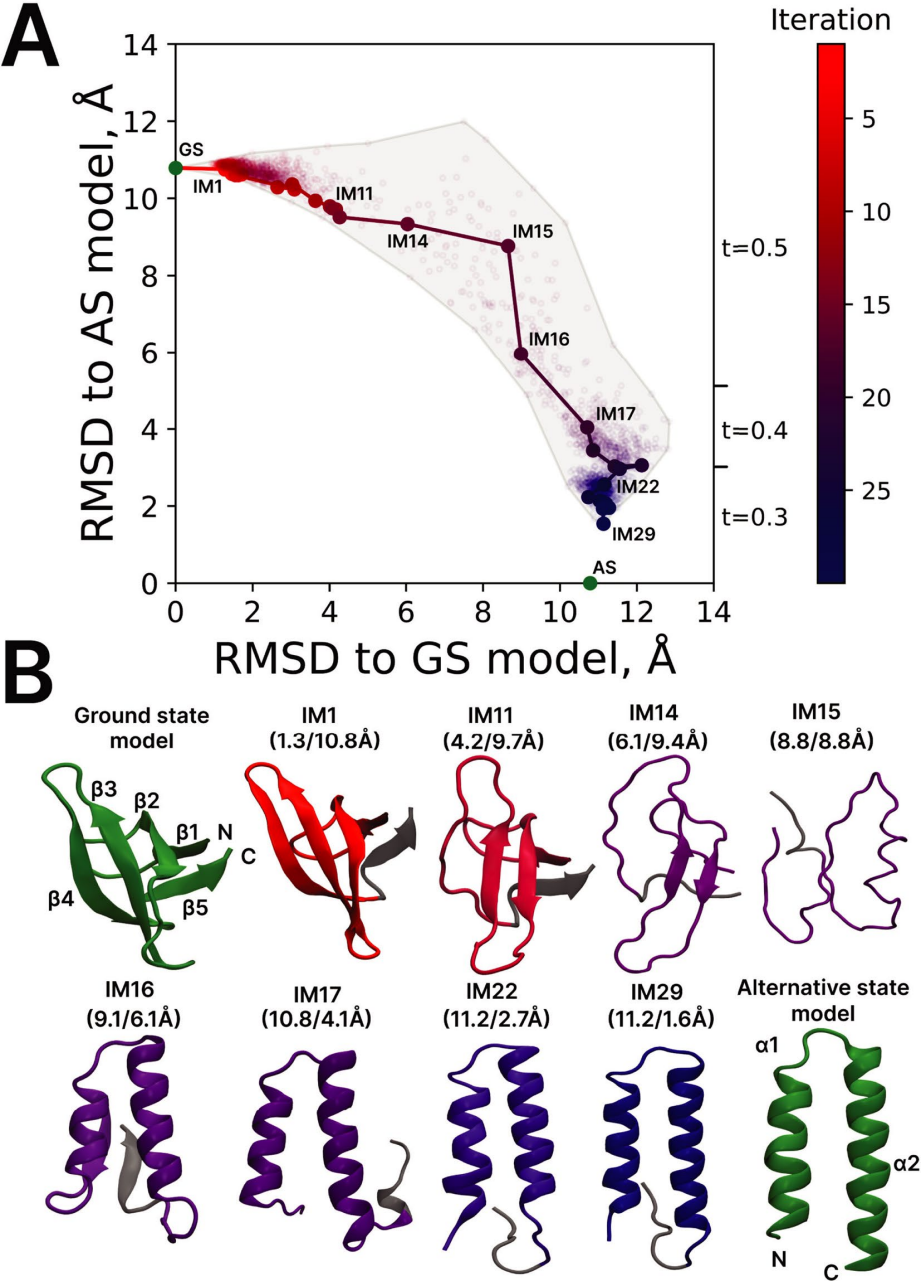
IMSD simulation of fold‐switching transition in RfaH‐CTD. (**A**) Conformational space map where each structural model is mapped according to its backbone *rmsd* relative to the GS and AS models. (**B**) Cartoon representation of GS and AS models (green), as well as selected IM models (colored from red to blue), representing the key transformations on the path from GS to AS. The portion of the structures that is not included in the calculations of *rmsd*, residues 157–162, is painted gray. The *rmsd* values relative to GS and AS models (calculated for residues 115–156) are indicated in brackets above each IM model. The secondary‐structure elements in the reference structures and their constituent residues are as follows: GS—β1 (116–118), β2 (126–130), β3 (138–144), β4 (149–153), β5 (159–161), AS—α1 (117–130), α2 (135–155). The evolution of the secondary structure throughout the trajectory is visualized in Figure S3C.

The IMSD trajectory for RfaH‐CTD is illustrated in Figure 4. The simulation uses the same default protocol as initially developed for GA98 (see Section 2.2). The first iteration leads to IM1 in close agreement with the initial GS model. The system then gradually drifts away from the ground state; by the time it reaches IM11, it has lost the edge strand β4 and the adjacent strand β3 has become shorter. After a few more iterations, only a short β2‐β3 segment survives from the original β‐barrel arrangement; the topology is also changed—the domain now resembles a twisted hairpin. The topology is further altered in going from IM14 to IM15 (note also the change in perspective in Figure 4B). The latter model has no identifiable secondary structure, although the nucleation of α2 helix is visible (residues 137–145) and, to a lesser extent, the nucleation of α1 can also be seen (residues 121–127).

Beginning with almost structureless IM15, the system starts to refold into an alternative conformation. At this point, the trajectory takes a turn and moves downward, reducing *rmsd* to the AS model (see Figure 4A). Already in IM16, we observe a partially formed αα‐hairpin comprised of residues 124 to 149. Interestingly, this αα‐hairpin is capped with β1‐β5 pairing on the back side. This arrangement is known as βααβ unit and can be found, for example, in metamorphic protein KaiB [66]. There is no reason to think that IM16 represents any significant intermediate, but such species may indeed occur in the process of GS‐to‐AS transition in RfaH‐CTD. In the next iteration, IM17, this distinctive arrangement disappears, giving way to the expanded αα‐hairpin (residues 120–151). After twelve additional rounds of refinement, the two helices become aligned, and the hairpin expands further, spanning residues 117 to 153. The final state, IM29, resembles closely the target AS model; see Figure 4B.

For systems like RfaH‐CTD, where the two folds are entirely different, the transition pathway likely involves (near) complete unfolding of the protein followed by refolding into a different structure. In this sense, it may not be accidental that the IM15 state in our IMSD simulation is essentially structureless (in contrast to GA98 and SA1 V90T intermediates which all retain significant elements of structure). At the same time, it should be noted that our IMSD approach focuses on intermediate states that generally tend to be more compact and partially structured while leaving out transition states that are more extended and disordered (see Concluding remarks for further discussion).

How does all of this correlate with the experimental evidence on RfaH‐CTD fold‐switching behavior? Zuber et al. have recently measured ^h3^ *J*_NC’_ couplings across hydrogen bonds in RfaH‐CTD [67]. By comparing the magnitudes of the coupling constants, they surmised that β2‐β3 pairing is the most stable portion of the β‐sheet. This is consistent with our simulations, which suggest that β4 is the first strand to experience unfolding, while β2‐β3 survive for some time.

Furthermore, their CEST measurements supplemented with urea titration data indicated that the sample of RfaHCTD contains ca. 5% of minor species, which are unfolded, but have two regions with helical propensity: 127–131 and especially 136–150 [67]. Characteristically, these regions feature elevated (15)N *R*_2_ relaxation rates, likely from helix‐coil exchange. These observations are in line with our results whereby the region 137–145 has been identified as the α2 nucleation site. This is also consistent with predictions by NetCSSP [68] and other programs that report strong helical propensity for the leucine‐rich motif _138_RSMLLLNLI_146_ within this region. All of this leads us to suggest that IM15 conformation observed in our simulations is representative of Uα species described by Zuber and co‐workers [67].

## 3 Concluding Remarks

Twelve years ago, Harada and Kitao proposed their Parallel cascade selection molecular dynamics (PaCS‐MD) scheme to explore conformational transition pathways [69]. The method assumes that there are two distinct conformational states, A and B, whose structures are known a priori. A series of short MD simulations is started from the structure A (using randomized initial velocities). The generated snapshots are ranked according to their *rmsd* relative to the target structure B (lower *rmsd* is better). The top‐ranking snapshots are then used to start the next series of short simulations. The process is iterated until highly ranked snapshots come sufficiently close to the conformation B. Using the PaCS‐MD method, Harada and Kitao have successfully modeled the transition between open and closed forms of T4‐lysozyme and also managed to fold a 10‐residue mini‐protein chignolin [69]. The attraction of the PaCS‐MD scheme is that it does not interfere with the native MD algorithm, i.e., it does not introduce any kind of biasing or steering potential to drive the system toward the state B. Instead, it steers the system in a milder way by selecting the conformers that are closest to B and using them to reseed the simulations.

The IMSD method proposed in this paper is conceptually similar to the PaCS‐MD scheme by Harada and Kitao. The obvious difference is that in our protocol, the structure is evolved through the generative diffusion algorithm (noising‐denoising steps) instead of the conventional MD run. The amount of noising (i.e., the magnitude of structural perturbation to the system) can be arbitrarily large, which means that IMSD can scale high energy barriers unlike the original PaCS‐MD scheme. This makes the IMSD protocol suitable for modeling major structural rearrangements such as metamorphic transitions.

Let us now compare IMSD to those Molecular Dynamics methods that are used to model fold‐switching transitions. For example, in the cases of GA98/GB98 and RfaH‐CTD, a number of modeling methods have been used, including different flavors of targeted MD, replica‐exchange MD, metadynamics, MD employing special structure‐based potentials, and a host of various hybrid schemes [16, 17, 34, 38, 51, 70, 71, 72]. A priori, we do not expect that these methods can faithfully reproduce the transition pathways in all their complexity. Indeed, current force fields experience issues even with the modeling of globular proteins [73]. It is clearly much harder to reproduce fold‐switching intermediates for a metamorphic protein. The main measure of success for such simulations is their agreement with the experimental data (e.g., NMR data on unfolding/refolding pathways in a given MP). Another question faced by MD methods is the question of statistics. While the simulation may correctly identify some of the key intermediates, it may miss some others.

Similar criticism can be directed at our IMSD scheme. The key premise of our approach is that the latest‐generation predictors, such as the AF2‐based generative diffusion algorithm UFConf, can meaningfully predict not only the fully folded protein forms, but also the partially folded intermediates. In turn, this implies that AF2 and its peer programs have learned the general principles of protein architecture. Although this seems to be the prevailing opinion in the field [74, 75, 76], some reservations have also been expressed [23, 77].

Note also that the IMSD approach is not intended to capture fold‐switching transition states, which are largely (although not entirely) disordered, extremely low‐populated, and short‐lived. Rather, it aims to model the intermediates, which are partially structured, can be substantially populated, and have longer lifetimes [78]. Indeed, the UFConf algorithm at the core of our scheme first disassembles the protein and then attempts to fold it, thus arriving at partially folded (i.e., stabilized) species. It is worth noting that, unlike transition states, folding intermediates lend themselves to direct experimental observation [79]. It is also evident that our IMSD trajectories in their current form do not provide a statistically complete description of fold‐switching pathways. Note, however, that IMSD simulations are sufficiently fast compared to most accelerated MD algorithms (see Methods). Therefore, it should be fairly straightforward to improve the statistical aspect of IMSD simulations.

Clearly, IMSD scheme can be parameterized in many different ways. In particular, instead of *rmsd* one can use other collective variables such as GDT‐HA, TM score or a fraction of native contacts Q [80, 81, 82]. We have explored the latter option and found that Q‐based IMSD schemes can successfully reproduce metamorphic transitions in some cases, but not in others. The results from Q‐based IMSD simulation of GA98 are fundamentally similar to those discussed above (see Supporting Information for details).

It should also be mentioned that UFConf is equipped with two original algorithms to simulate conformational transitions. The first algorithm is the Langevin dynamics. Briefly, the simulation starts from a noising step with t = 0.6. After that the system is evolved through a sequence of small noising‐denoising steps, resulting in a quasi‐continuous trajectory. While Fan et al. [29] have demonstrated that this algorithm can successfully simulate domain dynamics in adenylate kinase, it is not suited to cross any substantial energy barriers. We have recorded a trajectory of GA98 consisting of 5000 steps of Langevin dynamics; this simulation took 3 days but, expectably, showed no signs of fold‐switching transition.

The second algorithm by the developers of UFConf is Structural interpolation. This algorithm first generates noised versions of conformations A and B. These noised models, A′ and B′, are then weight‐averaged, and the resulting mixture is denoised, thus leading to a conformation somewhere on the transition path. By varying the proportion of A′ and B′ in the mixture, from 1 to 0, one presumably can sample the entire path from A to B irrespective of the height of the intervening energy barrier. Using this method, as implemented in UFConf, we were able to trace the fold‐switching transition in SA1 V90T. However, the algorithm failed our negative‐control test, refolding SA1 V90T into an unrelated HIV‐1 protease fold (see Supporting Information for details).

In conclusion, there is obviously room for different GDM‐based algorithms to model fold‐switching transitions in metamorphic proteins. Yet, at this point, our *rmsd*‐driven IMSD scheme appears to produce the best results. In our hands, the IMSD algorithm has constructed reasonable models of fold‐switching transitions in GA98 and RfaH‐CTD, consistent with the characteristic dual‐funnel shape of the folding landscape. Crucially, these models are in agreement with the available experimental data. In the case of SA1 V90T, our results apparently represent the first detailed model of the fold‐switching transition in this metamorphic protein. This model can be regarded as a theoretical prediction awaiting experimental verification.

In the future, it would be interesting to see if similar algorithms can be developed to model pathways of metamorphic evolution (i.e., evolution of one fold into another through accumulated sequence change). For certain protein families such as Cro there is evidence that evolutionary changes lead to structures that resemble early fold‐switching intermediates [83]. We envision that the IMSD scheme may have a role to play in exploring such transformations.

It is also conceivable that the IMSD algorithm can be generalized to model protein folding and unfolding, that is, transitions from disordered state A to structured state B and vice versa. In broad terms, IMSD can be viewed as a part of the emerging generation of modeling tools [22, 26] aiming to model protein dynamics by means of deep learning technology.

## 4 Methods

The choice of AS and GS models is described in the text and in the Supporting Information. In the case of the RfaH‐CTD GS model, we retain the core portion of the domain, residues 115 to 162. In doing so, we exclude the N‐terminal segment 108 to 114, which appears to be structured in the NMR model 2LCL, but is not a part of the β‐sheet and is displaced (becomes a linker) in the open form of full‐length RfaH, see structure 6C6S [84]. As a side note, this segment contains the _111_YPGD_114_ motif, which UFConf invariably models as a β‐turn. If included in the IMSD simulation, this β‐turn appears to stabilize the β1‐β2 hairpin in a number of states on the path from GS to AS. We believe that this is a modeling artifact, i.e., the propensity of this sequence to form a β‐turn is significantly exaggerated [85]. Therefore, we choose to exclude this fragment from the GS model, same as in the prior MD simulation studies [16, 38].

The IMSD protocol has been implemented using an in‐house python script, which is available for download (see Data availability statement). The default procedure, such as used for GA98, involves the following steps:

- The first round of UFConf calculations is launched using the input from .*json* file, which contains the following input parameters: filename for the starting structure (GS model), t (default setting 0.5), number of replicas (100), and the number of reverse diffusion steps (30).
- After the UFConf run is completed, all replicas are screened for steric clashes. The clashes are identified using a Biopython script by Abanades [86] with the default set of van der Waals radii and a default cutoff factor of 0.6. Replicas with more than 5 clashes are removed from consideration.
- The remaining replicas are characterized in terms of their backbone *rmsd* relative to the GS and AS models. The *rmsd* is calculated using the program MDAnalysis [87] for all backbone heavy atoms within the residue range as supplied by the user. The structure with the lowest *rmsd* to the AS model (termed IM) is saved. If the *rmsd* to the AS model is lower than 2 Å, the program terminates.
- A new .*json* file is generated which contains the filename for the starting structure (IM model from the previous step) and the t value which has been updated according to the following rule: t = 0.5 for *rmsd* ≥ 5 Å, t = 0.4 for 5 Å > *rmsd* ≥ 3 Å and t = 0.3 for *rmsd* < 3 Å. The requested number of replicas and the number of reverse diffusion steps remain unchanged. The next round of UFConf calculations is launched with these input parameters. The algorithm then cycles to step (ii).

If necessary, the above default protocol can be readily modified (such as described in Section 2.3). The IMSD trajectories for small proteins can be recorded in less than a day (18 h for 48‐residue domain RfaH‐CTD using NVIDIA GeForce RTX 3080 GPU card); for bigger proteins, the simulation takes a longer time (3 days for 95‐residue SA1 V90T using the same video card).

## Author Contributions

**Dmitrii A. Luzik:** conceptualization, investigation, writing – original draft, methodology, validation, visualization, software, formal analysis, data curation. **Nikolai R. Skrynnikov:** conceptualization, funding acquisition, writing – review and editing, writing – original draft, supervision, methodology.

## Supporting information


**Figure S1:** Single‐run UFConf predictions for metamorphic protein GB98.
**Figure S2:** Single‐run UFConf predictions for five different metamorphic proteins.
**Figure S3:** Secondary‐structure evolution in the IMSD trajectories of GA98, SA1 V90T and RfaH‐CTD.
**Figure S4:** Duplicate IMSD simulation of GA98 fold‐switching transition.
**Figure S5:** Reverse IMSD simulation for GA98 transition from the AS state to GS.
**Figure S6:** Negative‐control IMSD simulation for GA98.
**Figure S7:** Negative‐control IMSD simulation for non‐metamorphic protein ubiquitin.
**Figure S8:** Cartoon representation of full‐length RfaH in closed and open conformations.
**Appendix S1:** Alternative choice of collective variable: fraction of native contacts *Q*.
**Figure S9:** IMSD simulation of fold‐switching transition in GA98 using alternative distance measure.
**Figure S10:** IMSD simulation of fold‐switching transition in SA1 V90T using alternative distance measure.
**Appendix S2:** Structural interpolation algorithm and its application to SA1 V90T.
**Figure S11:** Structural interpolation simulation of fold‐switching transition in SA1 V90T.
**Figure S12:** Structural interpolation simulation of fold‐switching transition in SA1 V90T using decoy AS model.

## Data Availability

The data that support the findings of this study are openly available in Zenodo at https://doi.org/10.5281/zenodo.15068452, reference number 15068452.

## References

[1] J. C. Kendrew , G. Bodo , H. M. Dintzis , R. G. Parrish , H. Wyckoff , and D. C. Phillips , “A Three‐Dimensional Model of the Myoglobin Molecule Obtained by X‐Ray Analysis,” Nature 181, no. 4610 (1958): 662–666, 10.1038/181662a0. 13517261

[2] A. K. Dunker , J. D. Lawson , C. J. Brown , et al., “Intrinsically Disordered Protein,” Journal of Molecular Graphics & Modelling 19, no. 1 (2001): 26–59, 10.1016/S1093-3263(00)00138-8. 11381529

[3] E. S. Kuloglu , D. R. McCaslin , J. L. Markley , and B. F. Volkman , “Structural Rearrangement of Human Lymphotactin, a C Chemokine, Under Physiological Solution Conditions,” Journal of Biological Chemistry 277, no. 20 (2002): 17863–17870. 11889129 10.1074/jbc.M200402200 PMC4451178

[4] K. Madhurima , B. Nandi , and A. Sekhar , “Metamorphic Proteins: The Janus Proteins of Structural Biology,” Open Biology 11, no. 4 (2021): 210012, 10.1098/rsob.210012. 33878950 PMC8059507

[5] M. Das , N. Chen , A. LiWang , and L.‐P. Wang , “Identification and Characterization of Metamorphic Proteins: Current and Future Perspectives,” Biopolymers 112, no. 10 (2021): e23473, 10.1002/bip.23473. 34528703

[6] P. A. Alexander , Y. He , Y. Chen , J. Orban , and P. N. Bryan , “A Minimal Sequence Code for Switching Protein Structure and Function,” Proceedings of the National Academy of Sciences of the United States of America 106, no. 50 (2009): 21149–21154, 10.1073/pnas.0906408106. 19923431 PMC2779201

[7] B. M. Burmann , S. H. Knauer , A. Sevostyanova , et al., “An α Helix to β Barrel Domain Switch Transforms the Transcription Factor RfaH Into a Translation Factor,” Cell 150, no. 2 (2012): 291–303, 10.1016/j.cell.2012.05.042. 22817892 PMC3430373

[8] A. K. Kim and L. L. Porter , “Functional and Regulatory Roles of Fold‐Switching Proteins,” Structure 29, no. 1 (2021): 6–14, 10.1016/j.str.2020.10.006. 33176159 PMC8184262

[9] L. L. Porter and L. L. Looger , “Extant Fold‐Switching Proteins Are Widespread,” Proceedings of the National Academy of Sciences of the United States of America 115, no. 23 (2018): 5968–5973, 10.1073/pnas.1800168115. 29784778 PMC6003340

[10] A. F. Dishman , R. C. Tyler , J. C. Fox , et al., “Evolution of Fold Switching in a Metamorphic Protein,” Science 371, no. 6524 (2021): 86–90, 10.1126/science.abd8700. 33384377 PMC8017559

[11] J. W. Schafer and L. L. Porter , “Evolutionary Selection of Proteins With Two Folds,” Nature Communications 14, no. 1 (2023): 5478, 10.1038/s41467-023-41237-2. PMC10482954 37673981

[12] R. L. Tuinstra , F. C. Peterson , S. Kutlesa , E. S. Elgin , M. A. Kron , and B. F. Volkman , “Interconversion Between Two Unrelated Protein Folds in the Lymphotactin Native State,” Proceedings of the National Academy of Sciences of the United States of America 105, no. 13 (2008): 5057–5062, 10.1073/pnas.0709518105. 18364395 PMC2278211

[13] Y. G. Chang , S. E. Cohen , C. Phong , et al., “Circadian Rhythms. A Protein Fold Switch Joins the Circadian Oscillator to Clock Output in Cyanobacteria,” Science 349, no. 6245 (2015): 324–328, 10.1126/science.1260031. 26113641 PMC4506712

[14] C. Ghosh and B. Jana , “Curious Case of MAD2 Protein: Diverse Folding Intermediates Leading to Alternate Native States,” Journal of Physical Chemistry. B 126, no. 9 (2022): 1904–1916, 10.1021/acs.jpcb.2c00382. 35230837

[15] I. Artsimovitch and C. A. Ramírez‐Sarmiento , “Metamorphic Proteins Under a Computational Microscope: Lessons From a Fold‐Switching RfaH Protein,” Computational and Structural Biotechnology Journal 20 (2022): 5824–5837, 10.1016/j.csbj.2022.10.024. 36382197 PMC9630627

[16] J. B. Gc , Y. R. Bhandari , B. S. Gerstman , and P. P. Chapagain , “Molecular Dynamics Investigations of the α‐Helix to β‐Barrel Conformational Transformation in the RfaH Transcription Factor,” Journal of Physical Chemistry B 118, no. 19 (2014): 5101–5108, 10.1021/jp502193v. 24758259

[17] S. Parui , E. Brini , and K. A. Dill , “Computing Free Energies of Fold‐Switching Proteins Using MELD × MD,” Journal of Chemical Theory and Computation 19, no. 19 (2023): 6839–6847, 10.1021/acs.jctc.3c00679. 37725050

[18] M. Rivera , P. Galaz‐Davison , I. Retamal‐Farfán , E. A. Komives , and C. A. Ramírez‐Sarmiento , “Dimer Dissociation Is a Key Energetic Event in the Fold‐Switch Pathway of KaiB,” Biophysical Journal 121, no. 6 (2022): 943–955, 10.1016/j.bpj.2022.02.012. 35151633 PMC8943816

[19] J. Jumper , R. Evans , A. Pritzel , et al., “Highly Accurate Protein Structure Prediction With AlphaFold,” Nature 596, no. 7873 (2021): 583–589, 10.1038/s41586-021-03819-2. 34265844 PMC8371605

[20] D. Chakravarty and L. L. Porter , “AlphaFold2 Fails to Predict Protein Fold Switching,” Protein Science 31, no. 6 (2022): e4353, 10.1002/pro.4353. 35634782 PMC9134877

[21] H. K. Wayment‐Steele , A. Ojoawo , R. Otten , et al., “Predicting Multiple Conformations via Sequence Clustering and AlphaFold2,” Nature 625, no. 7996 (2024): 832–839, 10.1038/s41586-023-06832-9. 37956700 PMC10808063

[22] Y. Kalakoti and B. Wallner , “AFsample2: Predicting Multiple Conformations and Ensembles With AlphaFold2,” *bioRxiv* (2024): 2024.05.28.596195, 10.1101/2024.05.28.596195. PMC11882827 40045015

[23] D. Chakravarty , J. W. Schafer , E. A. Chen , et al., “AlphaFold Predictions of Fold‐Switched Conformations Are Driven by Structure Memorization,” Nature Communications 15, no. 1 (2024): 7296, 10.1038/s41467-024-51801-z. PMC11344769 39181864

[24] J. L. Watson , D. Juergens , N. R. Bennett , et al., “De Novo Design of Protein Structure and Function With RFdiffusion,” Nature 620, no. 7976 (2023): 1089–1100, 10.1038/s41586-023-06415-8. 37433327 PMC10468394

[25] J. B. Ingraham , M. Baranov , Z. Costello , et al., “Illuminating Protein Space With a Programmable Generative Model,” Nature 623, no. 7989 (2023): 1070–1078, 10.1038/s41586-023-06728-8. 37968394 PMC10686827

[26] K. E. Wu , K. K. Yang , R. van den Berg , et al., “Protein Structure Generation via Folding Diffusion,” Nature Communications 15, no. 1 (2024): 1059, 10.1038/s41467-024-45051-2. PMC10844308 38316764

[27] S. Zheng , J. He , C. Liu , et al., “Predicting Equilibrium Distributions for Molecular Systems With Deep Learning,” Nature Machine Intelligence 6, no. 5 (2024): 558–567, 10.1038/s42256-024-00837-3.

[28] M. Baek , F. DiMaio , I. Anishchenko , et al., “Accurate Prediction of Protein Structures and Interactions Using a Three‐Track Neural Network,” Science 373, no. 6557 (2021): 871–876, 10.1126/science.abj8754. 34282049 PMC7612213

[29] J. Fan , Z. Li , E. Alcaide , G. Ke , H. Huang , and E. Weinan , “Accurate Conformation Sampling via Protein Structural Diffusion,” Journal of Chemical Information and Modeling 64, no. 22 (2024): 8414–8426, 10.1021/acs.jcim.4c00928. 39340358

[30] P. A. Alexander , Y. He , Y. Chen , J. Orban , and P. N. Bryan , “The Design and Characterization of Two Proteins With 88% Sequence Identity but Different Structure and Function,” Proceedings of the National Academy of Sciences of the United States of America 104, no. 29 (2007): 11963–11968, 10.1073/pnas.0700922104. 17609385 PMC1906725

[31] P. N. Bryan and J. Orban , “Implications of Protein Fold Switching,” Current Opinion in Structural Biology 23, no. 2 (2013): 314–316, 10.1016/j.sbi.2013.03.001. 23518177

[32] P. Tian and R. B. Best , “Exploring the Sequence Fitness Landscape of a Bridge Between Protein Folds,” PLoS Computational Biology 16, no. 10 (2020): e1008285, 10.1371/journal.pcbi.1008285. 33048928 PMC7553338

[33] Y. He , Y. Chen , P. A. Alexander , P. N. Bryan , and J. Orban , “Mutational Tipping Points for Switching Protein Folds and Functions,” Structure 20, no. 2 (2012): 283–291, 10.1016/j.str.2011.11.018. 22325777 PMC3278708

[34] C. N. Song , Q. Wang , T. Xue , Y. Wang , and G. J. Chen , “Molecular Dynamics Simulations on the Conformational Transitions From the G_A_98 (G_A_88) to G_B_98 (G_B_88) Proteins,” Journal of Molecular Recognition 29, no. 12 (2016): 580–595, 10.1002/jmr.2558. 27480925

[35] N. A. Bernhardt , W. H. Xi , W. Wang , and U. H. E. Hansmann , “Simulating Protein Fold Switching by Replica Exchange With Tunneling,” Journal of Chemical Theory and Computation 12, no. 11 (2016): 5656–5666, 10.1021/acs.jctc.6b00826. 27767301

[36] D. Chakravarty , J. W. Schafer , E. A. Chen , J. R. Thole , and L. L. Porter , “AlphaFold2 Has More to Learn About Protein Energy Landscapes,” bioRxiv 12, no. 12 (2023): 571380, 10.1101/2023.12.12.571380.

[37] M. Kouza and U. H. E. Hansmann , “Folding Simulations of the A and B Domains of Protein G,” Journal of Physical Chemistry. B 116, no. 23 (2012): 6645–6653, 10.1021/jp210497h. 22214186 PMC3337360

[38] N. A. Bernhardt and U. H. E. Hansmann , “Multifunnel Landscape of the Fold‐Switching Protein RfaH‐CTD,” Journal of Physical Chemistry. B 122, no. 5 (2018): 1600–1607, 10.1021/acs.jpcb.7b11352. 29323497 PMC5823028

[39] V. Muñoz and L. Serrano , “Development of the Multiple Sequence Approximation Within the AGADIR Model of α‐Helix Formation: Comparison With Zimm‐Bragg and Lifson‐Roig Formalisms,” Biopolymers 41, no. 5 (1997): 495–509, 10.1002/(SICI)1097-0282(19970415)41. 9095674

[40] A. Drozdetskiy , C. Cole , J. Procter , and G. J. Barton , “JPred4: A Protein Secondary Structure Prediction Server,” Nucleic Acids Research 43, no. W1 (2015): W389–W394, 10.1093/nar/gkv332. 25883141 PMC4489285

[41] R. Yan , D. Xu , J. Yang , S. Walker , and Y. Zhang , “A Comparative Assessment and Analysis of 20 Representative Sequence Alignment Methods for Protein Structure Prediction,” Scientific Reports 3 (2013): 2619, 10.1038/srep02619. 24018415 PMC3965362

[42] A. Matouschek , J. T. Kellis , L. Serrano , and A. R. Fersht , “Mapping the Transition State and Pathway of Protein Folding by Protein Engineering,” Nature 340, no. 6229 (1989): 122–126, 10.1038/340122a0. 2739734

[43] R. Giri , A. Morrone , C. Travaglini‐Allocatelli , P. Jemth , M. Brunori , and S. Gianni , “Folding Pathways of Proteins With Increasing Degree of Sequence Identities but Different Structure and Function,” Proceedings of the National Academy of Sciences of the United States of America 109, no. 44 (2012): 17772–17776, 10.1073/pnas.1201794109. 22652570 PMC3497760

[44] F. J. Blanco , G. Rivas , and L. Serrano , “A Short Linear Peptide That Folds Into a Native Stable Beta‐Hairpin in Aqueous Solution,” Nature Structural Biology 1, no. 9 (1994): 584–590, 10.1038/nsb0994-584. 7634098

[45] J. Kuszewski , G. M. Clore , and A. M. Gronenborn , “Fast Folding of a Prototypic Polypeptide: The Immunoglobulin Binding Domain of Streptococcal Protein G,” Protein Science 3, no. 11 (1994): 1945–1952, 10.1002/pro.5560031106. 7703841 PMC2142643

[46] E. L. McCallister , E. Alm , and D. Baker , “Critical Role of β‐Hairpin Formation in Protein G Folding,” Nature Structural Biology 7, no. 8 (2000): 669–673, 10.1038/77971. 10932252

[47] I. A. Hubner , J. Shimada , and E. I. Shakhnovich , “Commitment and Nucleation in the Protein G Transition State,” Journal of Molecular Biology 336, no. 3 (2004): 745–761, 10.1016/j.jmb.2003.12.032. 15095985

[48] C. L. Brooks , “Protein and Peptide Folding Explored With Molecular Simulations,” Accounts of Chemical Research 35, no. 6 (2002): 447–454, 10.1021/ar0100172. 12069630

[49] P. Derreumaux , “Role of Supersecondary Structural Elements in Protein G Folding,” Journal of Chemical Physics 119, no. 9 (2003): 4940–4944, 10.1063/1.1596891.

[50] S. Kmiecik and A. Kolinski , “Folding Pathway of the B1 Domain of Protein G Explored by Multiscale Modeling,” Biophysical Journal 94, no. 3 (2008): 726–736, 10.1529/biophysj.107.116095. 17890394 PMC2186257

[51] T. Sikosek , H. Krobath , and H. S. Chan , “Theoretical Insights Into the Biophysics of Protein bi‐Stability and Evolutionary Switches,” PLoS Computational Biology 12, no. 6 (2016): e1004960, 10.1371/journal.pcbi.1004960. 27253392 PMC4890782

[52] M. Vidal , N. Goudreau , F. Cornille , D. Cussac , E. Gincel , and C. Garbay , “Molecular and Cellular Analysis of Grb2 SH3 Domain Mutants: Interaction With Sos and Dynamin,” Journal of Molecular Biology 290, no. 3 (1999): 717–730, 10.1006/jmbi.1999.2899. 10395825

[53] Y. Chen , Y. He , B. Ruan , et al., “Rules for Designing Protein Fold Switches and Their Implications for the Folding Code,” *bioRxiv* (2021): 2021.05.18.444643, 10.1101/2021.05.18.444643.

[54] B. Ruan , Y. He , Y. Chen , et al., “Design and Characterization of a Protein Fold Switching Network,” Nature Communications 14, no. 1 (2023): 431, 10.1038/s41467-023-36065-3. PMC9879998 36702827

[55] T. L. Solomon , Y. He , N. Sari , et al., “Reversible Switching Between Two Common Protein Folds in a Designed System Using Only Temperature,” Proceedings of the National Academy of Sciences of the United States of America 120, no. 4 (2023): e2215418120, 10.1073/pnas.2215418120. 36669114 PMC9942840

[56] Y. Shen , O. Lange , F. Delaglio , et al., “Consistent Blind Protein Structure Generation From NMR Chemical Shift Data,” Proceedings of the National Academy of Sciences of the United States of America 105, no. 12 (2008): 4685–4690. 18326625 10.1073/pnas.0800256105 PMC2290745

[57] B. Vallat , B. Webb , J. D. Westbrook , A. Sali , and H. M. Berman , “Development of a Prototype System for Archiving Integrative/Hybrid Structure Models of Biological Macromolecules,” Structure 26, no. 6 (2018): 894–904, 10.1016/j.str.2018.03.011. 29657133 PMC5990459

[58] M. Olofsson , S. Hansson , L. Hedberg , D. T. Logan , and M. Oliveberg , “Folding of S6 Structures With Divergent Amino Acid Composition: Pathway Flexibility Within Partly Overlapping Foldons,” Journal of Molecular Biology 365, no. 1 (2007): 237–248, 10.1016/j.jmb.2006.09.016. 17056063

[59] E. Haglund , J. Lind , T. Öman , A. Öhman , L. Mäler , and M. Oliveberg , “The HD‐Exchange Motions of Ribosomal Protein S6 Are Insensitive to Reversal of the Protein‐Folding Pathway,” Proceedings of the National Academy of Sciences of the United States of America 106, no. 51 (2009): 21619–21624, 10.1073/pnas.0907665106. 19966220 PMC2799792

[60] H. Lammert , J. K. Noel , E. Haglund , A. Schug , and J. N. Onuchic , “Constructing a Folding Model for Protein S6 Guided by Native Fluctuations Deduced From NMR Structures,” Journal of Chemical Physics 143, no. 24 (2015): 243141, 10.1063/1.4936881. 26723626

[61] P. K. Zuber , K. Schweimer , P. Rösch , I. Artsimovitch , and S. H. Knauer , “Reversible Fold‐Switching Controls the Functional Cycle of the Antitermination Factor RfaH,” Nature Communications 10, no. 1 (2019): 702, 10.1038/s41467-019-08567-6. PMC6370827 30742024

[62] S. K. Tomar , S. H. Knauer , M. Nandymazumdar , P. Rösch , and I. Artsimovitch , “Interdomain Contacts Control Folding of Transcription Factor RfaH,” Nucleic Acids Research 41, no. 22 (2013): 10077–10085, 10.1093/nar/gkt779. 23990324 PMC3905879

[63] A. C. Braisted and J. A. Wells , “Minimizing a Binding Domain From Protein A,” Proceedings of the National Academy of Sciences of the United States of America 93, no. 12 (1996): 5688–5692, 10.1073/pnas.93.12.5688. 8650153 PMC39121

[64] M. A. Starovasnik , A. C. Braisted , and J. A. Wells , “Structural Mimicry of a Native Protein by a Minimized Binding Domain,” Proceedings of the National Academy of Sciences of the United States of America 94, no. 19 (1997): 10080–10085, 10.1073/pnas.94.19.10080. 9294166 PMC23311

[65] A. A. Slavokhotova and E. A. Rogozhin , “Defense Peptides From the α‐Hairpinin Family Are Components of Plant Innate Immunity,” Frontiers in Plant Science 11 (2020): 465, 10.3389/fpls.2020.00465. 32391035 PMC7191063

[66] R. Pattanayek , D. R. Williams , S. Pattanayek , et al., “Structural Model of the Circadian Clock KaiB–KaiC Complex and Mechanism for Modulation of KaiC Phosphorylation,” EMBO Journal 27, no. 12 (2008): 1767–1778, 10.1038/emboj.2008.104. 18497745 PMC2435126

[67] P. K. Zuber , T. Daviter , R. Heißmann , U. Persau , K. Schweimer , and S. H. Knauer , “Structural and Thermodynamic Analyses of the β‐to‐α Transformation in RfaH Reveal Principles of Fold‐Switching Proteins,” eLife 11 (2022): e76630, 10.7554/eLife.76630. 36255050 PMC9683785

[68] C. Kim , J. Choi , S. J. Lee , W. J. Welsh , and S. Yoon , “NetCSSP: Web Application for Predicting Chameleon Sequences and Amyloid Fibril Formation,” Nucleic Acids Research 37 (2009): W469–W473, 10.1093/nar/gkp351. 19468045 PMC2703942

[69] R. Harada and A. Kitao , “Parallel Cascade Selection Molecular Dynamics (PaCS‐MD) to Generate Conformational Transition Pathway,” Journal of Chemical Physics 139, no. 3 (2013): 035103, 10.1063/1.4813023. 23883057

[70] S. Li , B. Xiong , Y. Xu , et al., “Mechanism of the all‐α to all‐β Conformational Transition of RfaH‐CTD: Molecular Dynamics Simulation and Markov State Model,” Journal of Chemical Theory and Computation 10, no. 6 (2014): 2255–2264, 10.1021/ct5002279. 26580748

[71] L. Xiong and Z. Liu , “Molecular Dynamics Study on Folding and Allostery in RfaH,” Proteins 83, no. 9 (2015): 1582–1592, 10.1002/prot.24839. 26033324

[72] B. Seifi and S. Wallin , “The C‐Terminal Domain of Transcription Factor RfaH: Folding, Fold Switching and Energy Landscape,” Biopolymers 112, no. 10 (2021): e23420, 10.1002/bip.23420. 33521926

[73] A. Raval , S. Piana , M. P. Eastwood , R. O. Dror , and D. E. Shaw , “Refinement of Protein Structure Homology Models via Long, All‐Atom Molecular Dynamics Simulations,” Proteins 80, no. 8 (2012): 2071–2079, 10.1002/prot.24098. 22513870

[74] J. P. Roney and S. Ovchinnikov , “State‐Of‐The‐Art Estimation of Protein Model Accuracy Using AlphaFold,” Physical Review Letters 129, no. 23 (2022): 238101, 10.1103/PhysRevLett.129.238101. 36563190 PMC12178128

[75] D. Sala , F. Engelberger , H. S. McHaourab , and J. Meiler , “Modeling Conformational States of Proteins With AlphaFold,” Current Opinion in Structural Biology 81 (2023): 102645, 10.1016/j.sbi.2023.102645. 37392556

[76] A. Aranganathan , X. Gu , D. Wang , B. P. Vani , and P. Tiwary , “Modeling Boltzmann‐Weighted Structural Ensembles of Proteins Using Artificial Intelligence–Based Methods,” Current Opinion in Structural Biology 91 (2025): 103000, 10.1016/j.sbi.2025.103000. 39923288 PMC12011212

[77] D. Chakravarty , M. Lee , and L. L. Porter , “Proteins With Alternative Folds Reveal Blind Spots in AlphaFold‐Based Protein Structure Prediction,” Current Opinion in Structural Biology 90 (2025): 102973, 10.1016/j.sbi.2024.102973. 39756261 PMC11791787

[78] R. L. Baldwin and G. D. Rose , “Is Protein Folding Hierarchic? II. Folding Intermediates and Transition States,” Trends in Biochemical Sciences 24, no. 2 (1999): 77–83, 10.1016/s0968-0004(98)01345-0. 10098403

[79] H. J. Dyson and P. E. Wright , “Equilibrium NMR Studies of Unfolded and Partially Folded Proteins,” Nature Structural Biology 5, no. Suppl (1998): 499–503, 10.1038/739. 9665178

[80] K. Olechnovič , B. Monastyrskyy , A. Kryshtafovych , and Č. Venclovas , “Comparative Analysis of Methods for Evaluation of Protein Models Against Native Structures,” Bioinformatics 35, no. 6 (2019): 937–944, 10.1093/bioinformatics/bty760. 30169622 PMC6419896

[81] Y. Zhang and J. Skolnick , “TM‐Align: A Protein Structure Alignment Algorithm Based on the TM‐Score,” Nucleic Acids Research 33, no. 7 (2005): 2302–2309, 10.1093/nar/gki524. 15849316 PMC1084323

[82] R. B. Best , G. Hummer , and W. A. Eaton , “Native Contacts Determine Protein Folding Mechanisms in Atomistic Simulations,” Proceedings of the National Academy of Sciences of the United States of America 110, no. 44 (2013): 17874–17879, 10.1073/pnas.1311599110. 24128758 PMC3816414

[83] V. K. Kumirov , E. M. Dykstra , B. M. Hall , W. J. Anderson , T. N. Szyszka , and M. H. J. Cordes , “Multistep Mutational Transformation of a Protein Fold Through Structural Intermediates,” Protein Science 27, no. 10 (2018): 1767–1779, 10.1002/pro.3488. 30051937 PMC6199151

[84] J. Y. Kang , R. A. Mooney , Y. Nedialkov , et al., “Structural Basis for Transcript Elongation Control by NusG Family Universal Regulators,” Cell 173, no. 7 (2018): 1650–1662, 10.1016/j.cell.2018.05.017. 29887376 PMC6003885

[85] A. V. Sarma , T. V. Raju , and A. C. Kunwar , “NMR Study of the Peptide Present in the Principal Neutralizing Determinant (PND) of HIV‐1 Envelope Glycoprotein gp120,” Journal of Biochemical and Biophysical Methods 34, no. 2 (1997): 83–98, 10.1016/s0165-022x(97)01205-0. 9178085

[86] Biopython Script to Check PDB Files for Clashing Atoms [Computer Program] (Oxford Protein Informatics Group, 2023).

[87] N. Michaud‐Agrawal , E. J. Denning , T. B. Woolf , and O. Beckstein , “MDAnalysis: A Toolkit for the Analysis of Molecular Dynamics Simulations,” Journal of Computational Chemistry 32, no. 10 (2011): 2319–2327, 10.1002/jcc.21787. 21500218 PMC3144279

