# Supplementary material for: Iterative Modeling via Structural Diffusion (IMSD): Exploring Fold‐Switching Pathways in Metamorphic Proteins Using AlphaFold2‐Based Generative Diffusion Model UFConf
Source: Proteins. 2025 Sep 24;94(2):633–48. doi: 10.1002/prot.70050 (PMC12779232; doi:10.1002/prot.70050)
Supplement: Supplementary file 1 — Figure S1: Single‐run UFConf predictions for metamorphic protein GB98. Figure S2: Single‐run UFConf predictions for five different metamorphic proteins. Figure S3: Secondary‐structure evolution in the IMSD trajectories of GA98, SA1 V90T and RfaH‐CTD. Figure S4: Duplicate IMSD simulation of GA98 fold‐switching transition. Figure S5: Reverse IMSD simulation for GA98 transition from the AS state to GS. Figure S6: Negative‐control IMSD simulation for GA98. Figure S7: Negative‐control IMSD simulation for non‐metamorphic protein ubiquitin. Figure S8: Cartoon representation of full‐length RfaH in closed and open conformations. Appendix S1: Alternative choice of collective variable: fraction of native contacts Q. Figure S9: IMSD simulation of fold‐switching transition in GA98 using alternative distance measure. Figure S10: IMSD simulation of fold‐switching transition in SA1 V90T using alternative distance measure. Appendix S2: Structural interpolation algorithm and its application to SA1 V90T. Figure S11: Structural interpolation simulation of fold‐switching transition in SA1 V90T. Figure S12: Structural interpolation simulation of fold‐switching transition in SA1 V90T using decoy AS model. [file PROT-94-633-s001.pdf]

SUPPLEMENTARY INFORMATION

Iterative modeling via structural diffusion (IMSD): exploring fold-switching pathways in metamorphic proteins using AlphaFold2-based generative diffusion model UFConf

Dmitrii A. Luzik[1] and Nikolai R. Skrynnikov[1,2*]

[1] *Laboratory of Biomolecular NMR, St. Petersburg State University, St. Petersburg, Russia*
[2] *Department of Chemistry, Purdue University, West Lafayette, Indiana, USA.*

* *E-mail: n.skrynnikov@spbu.ru*

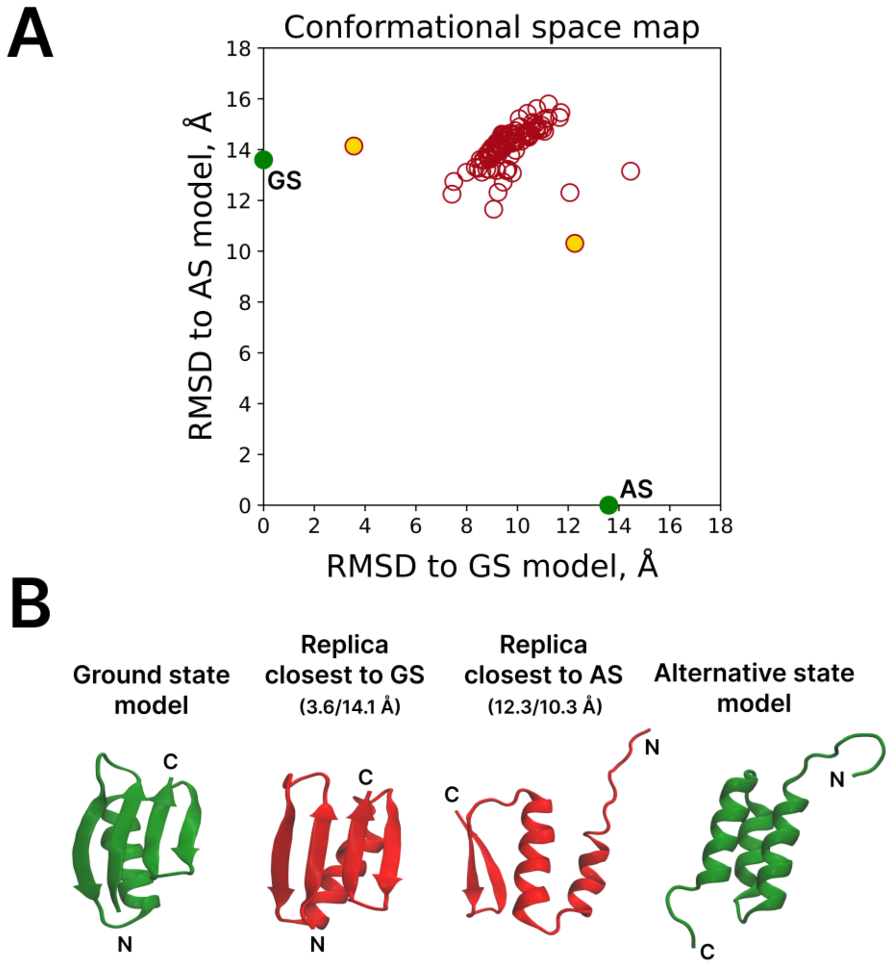

Figure S1. Single-run UFConf predictions for metamorphic protein GB98 (t = 1, 100 replicas). (**A)** Conformational space map where each replica is mapped according to its backbone *rmsd* relative to the pair of reference structures, GS (first model from the NMR-determined structure 2LHD) and AS (first model from the NMR-determined structure 2LHC). Gold-filled circles represent the replicas with lowest *rmsd* to the GS and AS models. (**B)** Cartoon representation of the GS and AS models (green), as well as UFConf replicas with lowest *rmsd* to the GS and AS models (red). For the UFConf replicas, the values of *rmsd* to the GS and AS models are indicated in brackets above the models.

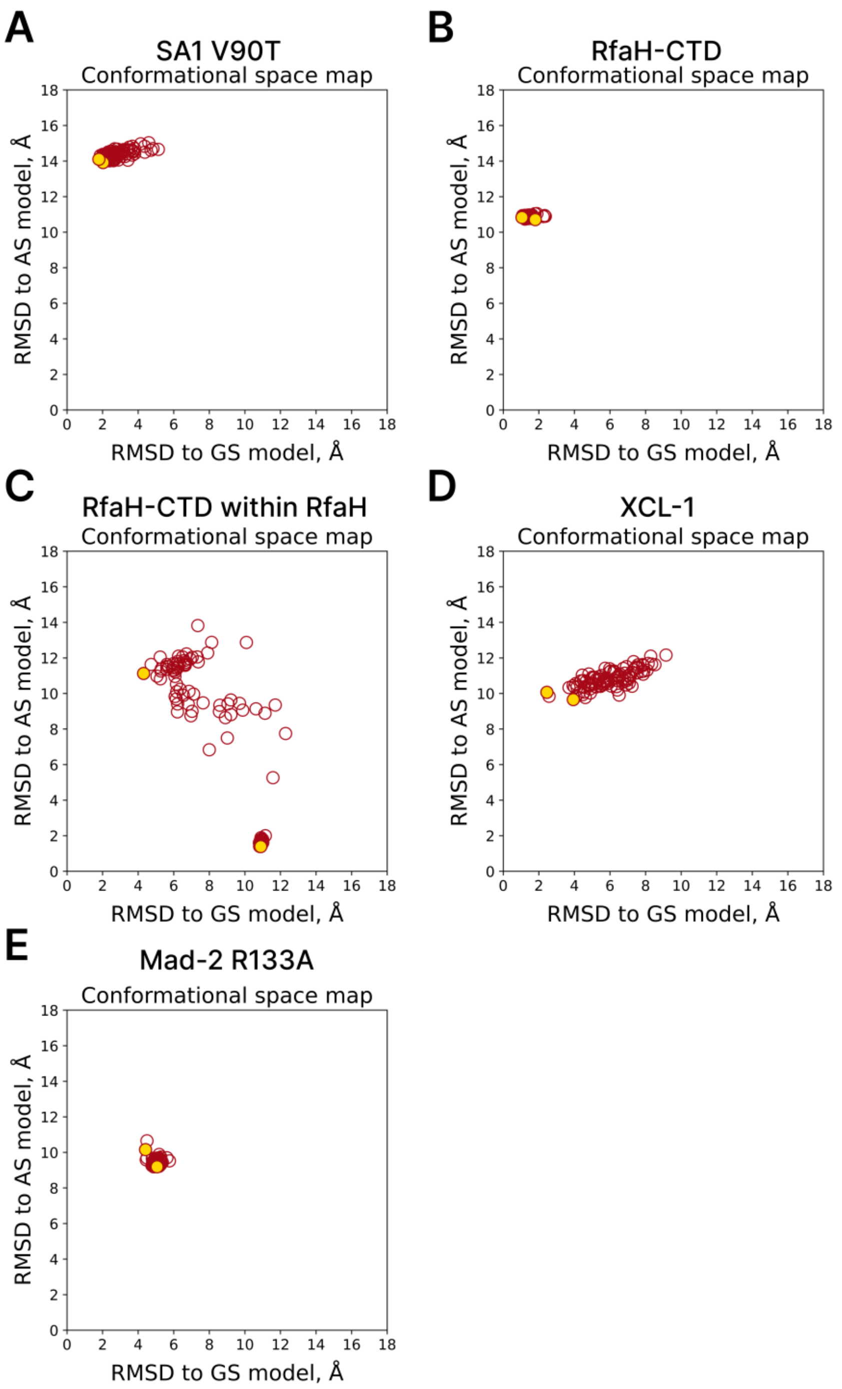

Figure S2. Single-run UFConf predictions for five different metamorphic proteins (t = 1, 100 replicas per run). The results are shown in a form of conformational space maps, where each replica is mapped according to its backbone *rmsd* relative to the pair of reference structures, GS and AS. Gold-filled circles represent the replicas with lowest *rmsd* to the GS and AS models. The maps are drawn for **(A)** SA1 V90T (GS PDB id 8E6Y, AS PDB-IHM id 9A20), **(B)** isolated C-terminal domain of RfaH (GS PDB id 2LCL: residues 115-162, AS PDB id 2OUG: residues 115-156), **(C)** C-terminal domain of RfaH within full-length RfaH (GS PDB id 6C6S: chain D, residues 115-156, AS PDB id 2OUG: residues 115-156), **(D)** XCL1 (GS PDB id 1J9O: residues 1:60, AS PDB id 2JP1: residues 1:60) and **(E)** Mad2 R133A (GS PDB id 1S2H: residues 11-195, AS PDB id 1DUJ: residues 13-197). For those structures that have been solved by solution NMR, the first model has been used in the simulations.

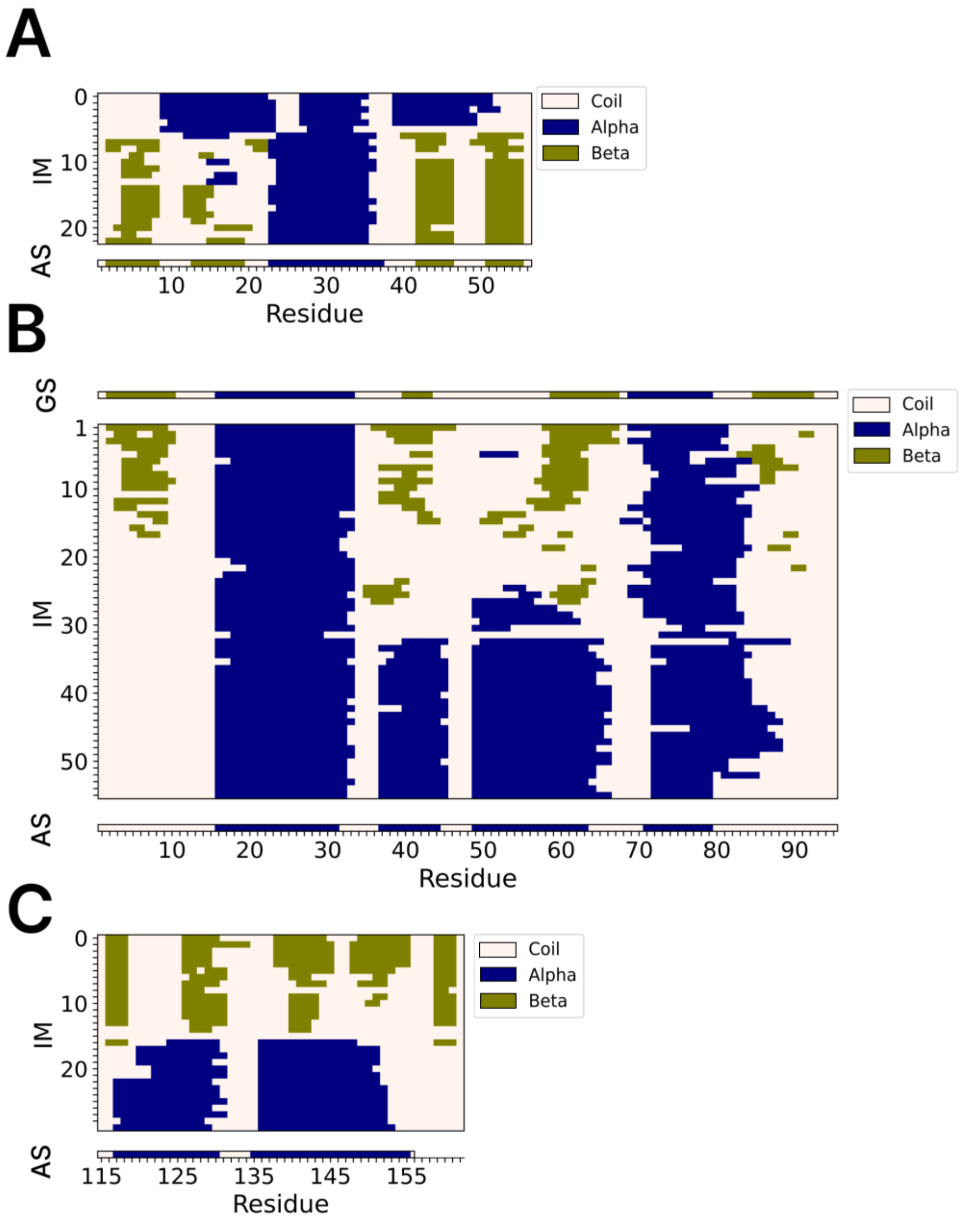

Figure S3. Secondary-structure evolution in the IMSD trajectories of metamorphic proteins (**A**) GA98, (**B**) SA1 V90T and (**C**) RfaH-CTD. The *y*-axis in the plot enumerates the series of consecutive IM states. In panels (A) and (C), the numbering starts with IM0 (GS model). In panel (B), GS is not considered a part of the IM series (see text) and, therefore, is represented by a separate stripe at the top of the plot. The AS model, which is never a part of the IM series, is represented by a stripe at the bottom of the plot. In panel (C), residues 157-162 are absent from the crystallographic-structure-derived AS model. The secondary-structure assignment has been obtained using the program STRIDE.[1]

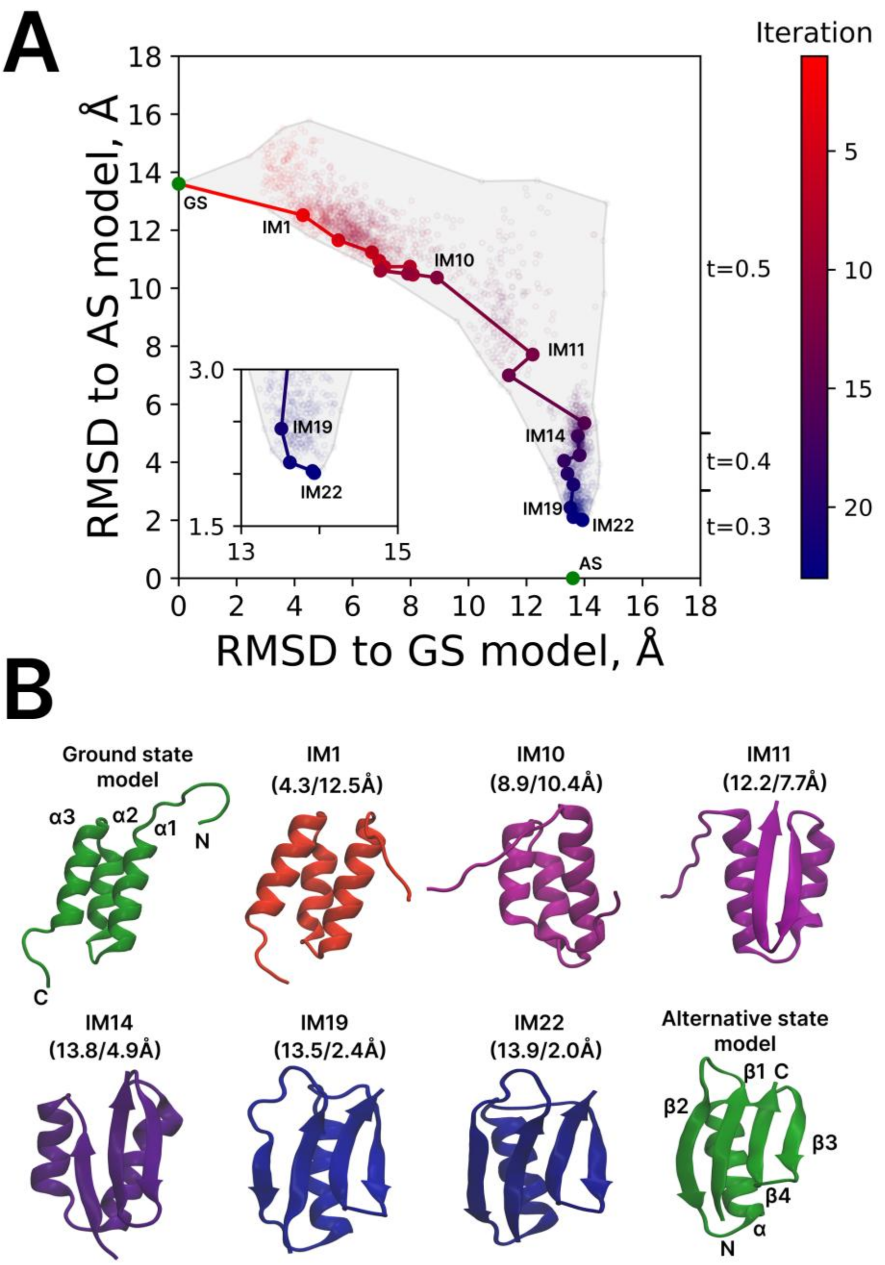

Figure S4. Duplicate IMSD simulation of GA98 fold-switching transition. (**A**) Conformational space map where each structural model is mapped according to its backbone *rmsd* relative to the GS and AS models. Bright solid circles represent the IM models, pale empty circles represent all other UFConf-generated replicas (colored from red to blue according to iteration number, cf. color bar on the right); bright green circles represents the GS state (starting model) and AS state (target). The grey shaded area is drawn around all replicas generated during the simulation. Shown in the insert is the magnified image of the final portion of the IMSD trajectory (iterations 19 to 22). The ranges of parameter t used for UFConf modeling are indicated on the right side of the plot. (**B**) Cartoon representation of GS and AS models (green), as well as selected IM models (colored from red to blue), representing the key transformations on the path from GS to AS. The *rmsd* values relative to GS and AS models are indicated in brackets above each IM model.

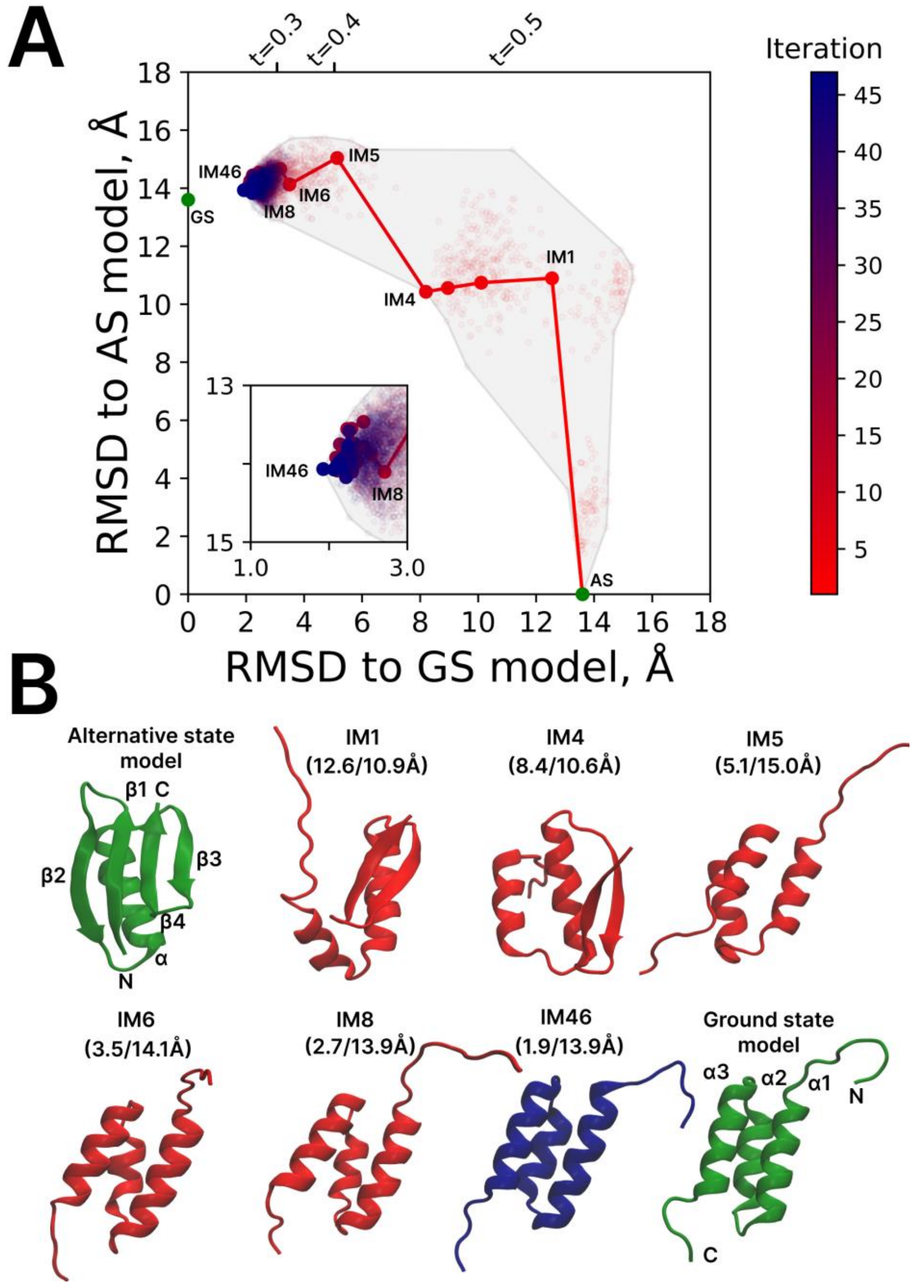

Figure S5. Reverse IMSD simulation to model the transition of GA98 from the AS state to GS state. The starting AS model has been generated by introducing the point mutation Y45L into the structure 2LHD using the program MODELLER 10.4.[2] The simulation protocol is the same as the one used to generate Fig. 2. (**A**) Conformational space map where each structural model is mapped according to its backbone *rmsd* relative to the GS and AS models. Bright solid circles represent the IM models, pale empty circles represent all other UFConf-generated replicas (colored from red to blue according to iteration number, cf. color bar on the right); bright green circles represents the AS state (starting model) and GS state (target). The grey shaded area is drawn around all replicas generated during the simulation. The ranges of parameter t used for UFConf modeling are indicated at the top of the plot. Similar rapidly progressing transitions from AS to GS were also observed for other metamorphic proteins investigated in this study (not shown). (**B**) Cartoon representation of AS and GS models (green), as well as selected IM models (colored from red to blue), representing the key transformations on the path from AS to GS. The *rmsd* values relative to GS and AS models are indicated in brackets above each IM model.

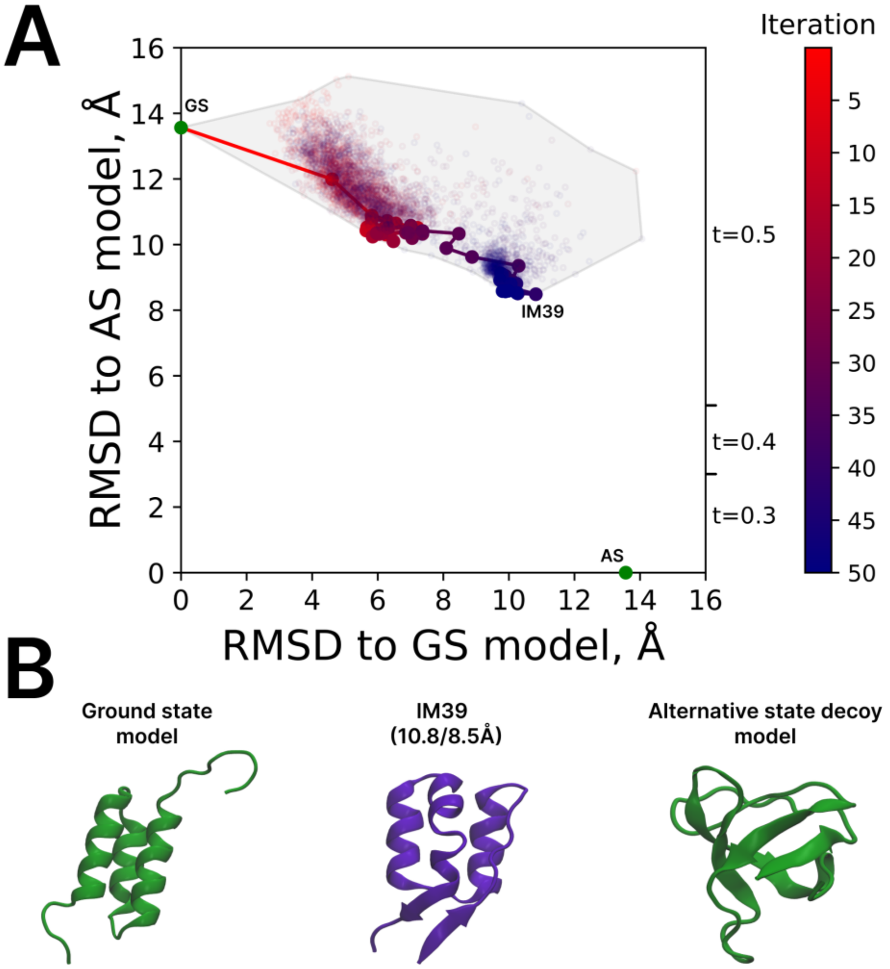

Figure S6. Negative-control IMSD simulation to explore possible transition of GA98 from the GS state (PDB id 2LHC) to the decoy AS state (56-residue N-terminal SH3 domain from adapter protein Grb2, PDB id 1AZE). The simulation protocol is the same as the one used to generate Fig. 2. (**A**) Conformational space map where each structural model is mapped according to its backbone *rmsd* relative to the GS and AS models. Bright solid circles represent the IM models, pale empty circles represent all other UFConf-generated replicas (colored from red to blue according to iteration number, cf. color bar on the right); bright green circles represents the GS state (starting model) and the decoy AS state (target). The grey shaded area is drawn around all replicas generated during the simulation. The ranges of parameter t used for UFConf modeling are indicated on the right side of the plot. The calculations have been terminated after 50 iterations. (**B**) Cartoon representation of GS and decoy AS models (green), as well as IM39 model (dark purple), which has the lowest *rmsd* to the decoy AS among all IM states. Note that all replicas generated in iteration 40 and subsequent iterations fall further away from the decoy AS model than IM39.

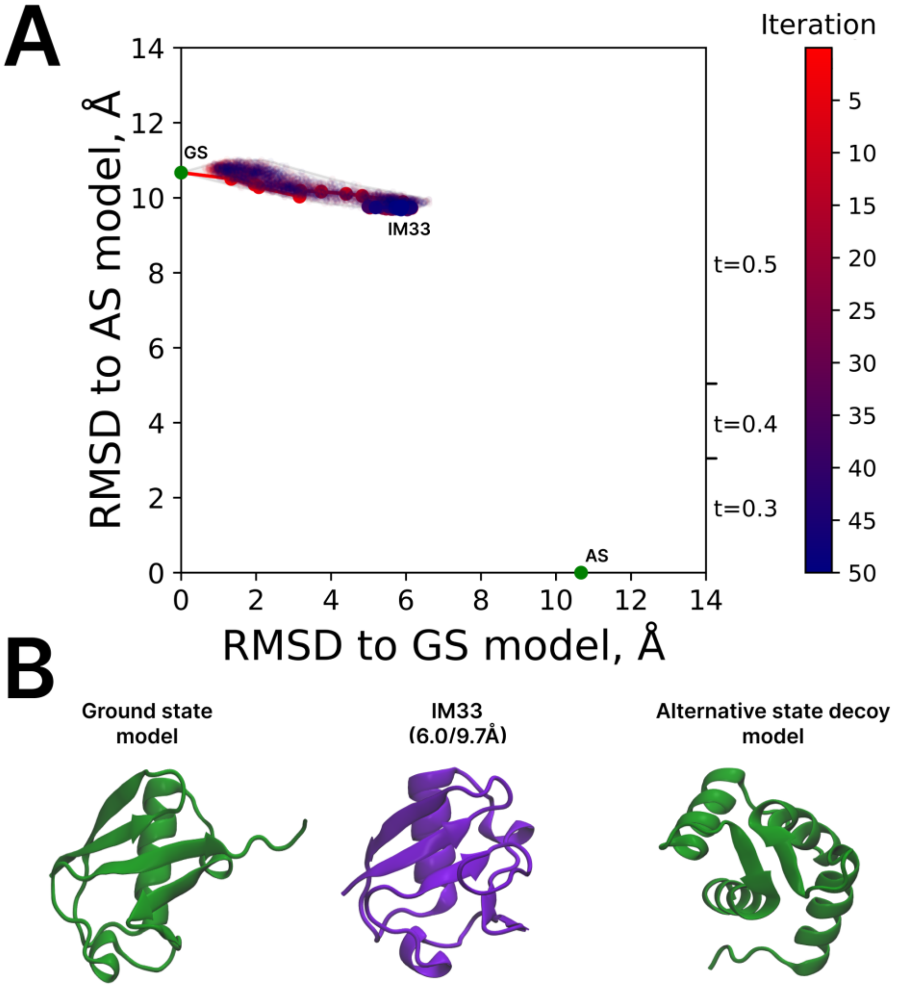

Figure S7. Negative-control IMSD simulation to explore possible transition of non-metamorphic protein ubiquitin from the GS state (PDB id 1UBQ) to the decoy AS state (76-residue N-terminal domain of calmodulin, PDB id 1F70). The simulation protocol is the same as the one used to generate Fig. 2. (**A**) Conformational space map where each structural model is mapped according to its backbone *rmsd* relative to the GS and AS models. Bright solid circles represent the IM models, pale empty circles represent all other UFConf-generated replicas (colored from red to blue according to iteration number, cf. color bar on the right); bright green circles represents the GS state (starting model) and the decoy AS state (target). The grey shaded area is drawn around all replicas generated during the simulation. The ranges of parameter *t* used for UFConf modeling are indicated on the right side of the plot. The calculations have been terminated after 50 iterations. (**B**) Cartoon representation of GS and decoy AS models (green), as well as IM33 model (purple), which has the lowest *rmsd* to the decoy AS among all IM states. Note that all replicas generated in iteration 34 and subsequent iterations fall further away from the decoy AS model than IM33.

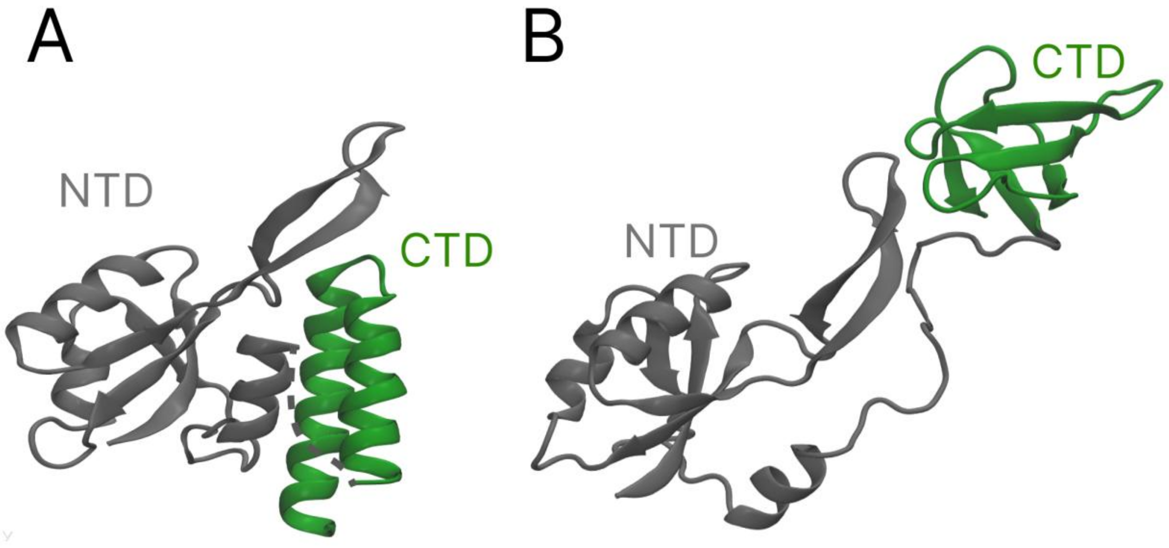

Figure S8. Cartoon representation of full-length RfaH from *E. coli* in (**A**) autoinhibited closed conformation (PDB id 2OUG, dashed line represents unresolved interdomain linker) and (**B**) active open conformation (PDB id 6C6S, other components of the complex except RfaH are not shown). The N- and C-terminal domains of RfaH are painted grey and green, respectively.

APPENDIX S1. Alternative choice of collective variable: fraction of native contacts Q

At the suggestion of the reviewer, we tested a variant of the IMSD scheme equipped with an alternative distance measure, i.e. replaced backbone *rmsd* with (inverse) fraction of native amino acid contacts Q. First, we turned to the widely used definition of Q introduced by Best *et al.*[3] However, our Q-based IMSD simulations performed on GA98 and SA1 V90T failed to leave the GS well. To resolve this problem we invoked a different definition as described by Duarte *et al.*:[4]

$$Q = \frac{N_{model}}{N_{ref}}$$

where it is assumed that a pair of C^{α} atoms within 10 Å of each other constitute a contact, N_{ref} is a number of contacts found in the reference structure (AS), and N_{model} is a number of contacts found in both UFConf-generated model (replica) and the reference structure. We expected that this definition should offer enough structural flexibility to generate more expanded/disordered IM species.

Using our original IMSD protocol as a starting point, we replaced the backbone *rmsd* variable with the inverse fraction of native contacts, $\lambda = 1/Q$. After some trials, we selected the following λ thresholds: λ = 1.4 (t is lowered from 0.5 to 0.4), λ = 1.2 (t is further lowered to 0.3) and λ = 1.1 (the simulation is terminated). Other details of the IMSD procedure were the same as described in the main text. Using this alternative setup, we have recorded the IMSD trajectory for GA98, as illustrated in Fig. S9.

Inspection of Fig. S9 shows that replacing backbone *rmsd* with inverse fraction of native contacts λ causes little change to the results. The transition begins with a loss of α3 helix (IM2) followed by formation of β3-β4 hairpin (IM3). After a few more iterations the nascent β1 strand emerges (cf. IM9). Finally, the surviving α1 helix is transformed into β2 strand at the edge of the newly formed β-sheet (cf. IM35). Simulation stops at IM41, where the protein adopts AS-like fold with λ = 1.1.

As it happens, the IM species generated during this simulation are no more disordered or expanded than those obtained previously, see Fig. 2. We thus observe that the new Q-based procedure simply reproduces (at the qualitative level) the earlier results obtained by means of the *rmsd*-based scheme.

After the successful GA98 simulation, we turned to SA1 V90T. The protocol was the same as previously employed for this system (including the use of *t* = 1 in the first iteration), but with λ in place of *rmsd*. λ thresholds were assigned as described above. Using this modified scheme, we have recorded the IMSD trajectory for SA1 V90T. As it turned out, though, after 50 iterations the simulation failed to leave the well in the vicinity of the GS state, see Fig. S10.

To address this issue, we attempted to change the t schedule and further modify the definition of collective variable (e.g. use a difference between the numbers of native and non-native contacts). However, these efforts led to mixed results: some of the simulations were successful, while others failed (not shown). We conclude that while alternative IMSD schemes employing contact-based measures are generally viable, currently the *rmsd*-based protocol appears to be more stable and efficient.

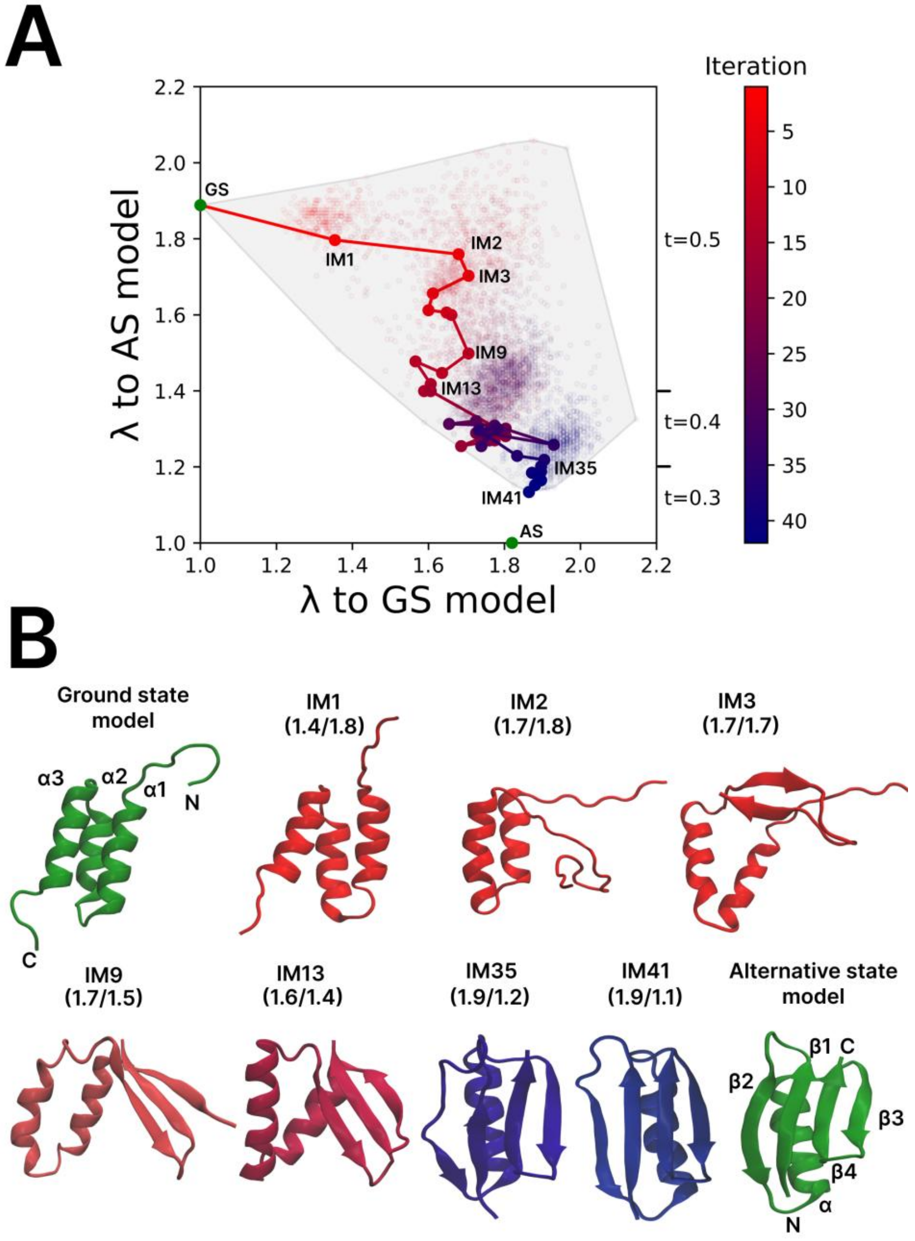

Figure S9. IMSD simulation of fold-switching transition in GA98 using alternative distance measure, λ. (**A**) Conformational space map where each structural model is mapped according to its (inverse) fraction of native contacts, λ, relative to the GS and AS models. (**B**) Cartoon representation of GS and AS models (green), as well as selected IM models (colored from red to blue), representing the key transformations on the path from GS to AS. The λ values relative to GS and AS models are indicated in brackets above each IM model. Other notations are the same as in Fig. 2.

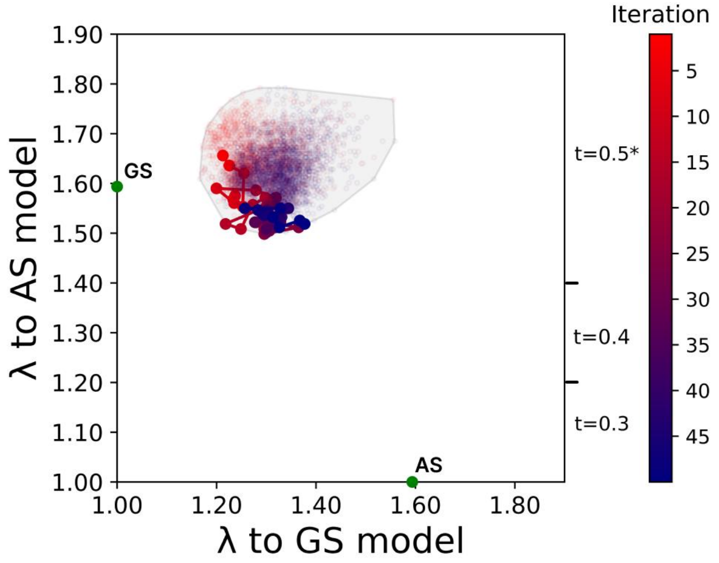

Figure S10. IMSD simulation of fold-switching transition in SA1 V90T using alternative distance measure, λ. The simulation did not show any significant progress toward AS and has been terminated after 50 iterations.

APPENDIX S2. Structural interpolation algorithm and its application to SA1 V90T

Structural interpolation algorithm has been proposed[5] as UFConf-based tool to model proteins conformational dynamics. Conceivably, it can also be used to model fold-switching transitions in metamorphic proteins. To explore this possibility, we tested structural interpolation method on SA1 V90T. The simulations were set up using the same protocol as originally reported. Specifically, GS and AS structures (PDB id 8E6Y and 9A20, respectively) have been noised with t = 0.3. The resulting two models were then interpolated on a 100-point grid and each of the interpolation models was reverse-diffused with 10 inference steps to recover structures on a path from GS to AS.

The results from the structural interpolation trajectory are shown in Fig. S11. As can be seen from the graph, the simulation produces distinctive linear pattern in the conformational space map. The linearity suggests that the results are largely a function of the initial conditions generated by the linear interpolation scheme. Nevertheless, the secondary-structure evolution map appears reasonable and reproduces the characteristic features previously observed in our IMSD simulations, e.g. the early loss of the β4 strand (cf. Fig. S3B).

To further validate the structural interpolation method we have set up a negative-control simulation along the lines of Fig. S7. Specifically, we have used an unrelated protein fold, that of mature HIV-1 protease,[6] as an AS decoy. Obviously, a valid algorithm should not transform the GS structure into an alternative state that represents a completely irrelevant backbone fold. Yet structural interpolation scheme does exactly that, see Fig. S12.

In attempt to remedy this situation we increased the parameter t in the structure interpolation protocol to 0.4 and then to 0.5. However, the results remained mixed: the algorithm performed reasonably for some MPs, but not for others. We conclude that the idea of structural interpolation can likely find some use in modeling of fold-switching transitions in metamorphic proteins, but at this point the *rmsd*-driven IMSD scheme appears to be preferable.

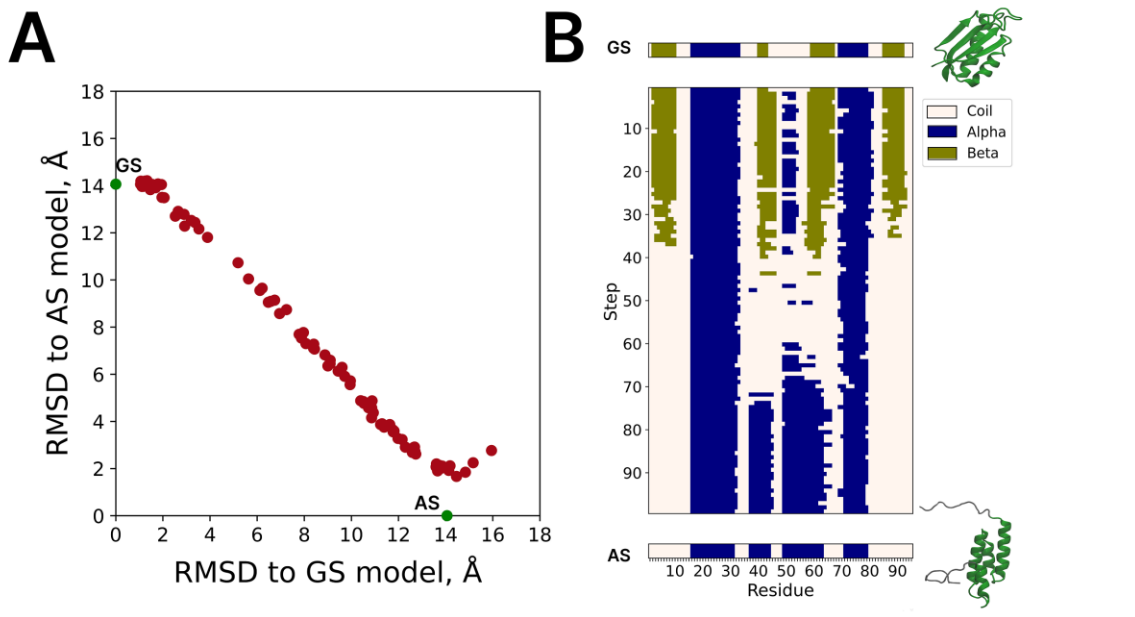

Figure S11. Structural interpolation simulation of fold-switching transition in SA1 V90T. (**A**) Conformational space map parameterized in terms of backbone *rmsd* relative to the GS structure (PDB id 8E6Y) and AS model (PDB-IHM id 9A20). (**B**) Time trace of the secondary-structure evolution; GS and AS structures are shown by the side of the plot (colored green).

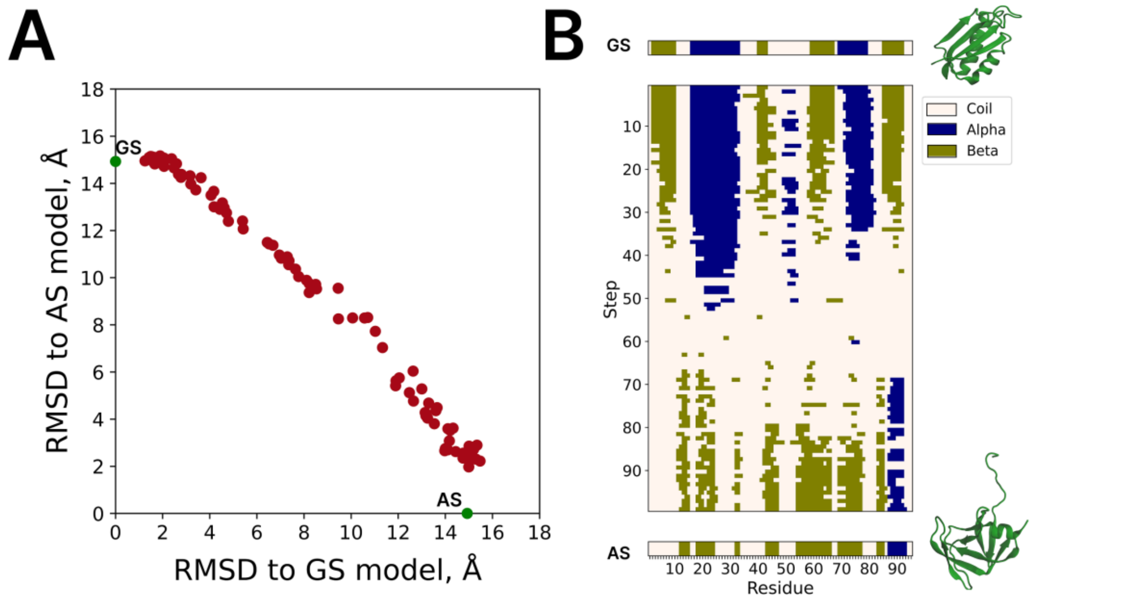

Figure S12. Structural interpolation simulation of fold-switching transition in SA1 V90T using decoy AS structure. The decoy is based on the coordinates of 95-residue monomer of mature HIV-1 protease (PDB id 1Q9P). The decoy model was generated through step-by-step replacement of the original protease residues with SA1 V90T residues using MODELLER 10.4. Since only the backbone is subjected to noising/denoising treatment in the original structural interpolation algorithm,[5] incorrect side chain packing and clashes resulting from *in silico* mutagenesis can be ignored. (**A**) Conformational space map parameterized in terms of backbone *rmsd* relative to the GS structure (PDB id 8E6Y) and decoy AS model. (**B**) Time trace of the secondary-structure evolution; GS and decoy AS structures are shown by the side of the plot (colored green).

SUPPLEMENTARY REFERENCES

1. Frishman D, Argos P. Knowledge-based protein secondary structure assignment. *Proteins: Struct, Funct, Genet.* 1995;23(4):566-579. doi:10.1002/prot.340230412
2. Webb B, Sali A. Comparative Protein Structure Modeling Using MODELLER. *Curr Protoc Bioinform.* 2016;54(1):5.6.1-5.6.37. doi:doi.org/10.1002/cpbi.3
3. Best RB, Hummer G, Eaton WA. Native contacts determine protein folding mechanisms in atomistic simulations. *Proc Natl Acad Sci USA.* 2013;110(44):17874-17879. doi:doi:10.1073/pnas.1311599110
4. Duarte JM, Sathyapriya R, Stehr H, Filippis I, Lappe M. Optimal contact definition for reconstruction of contact maps. *BMC Bioinformatics.* 2010;11:283. doi:10.1186/1471-2105-11-283
5. Fan J, Li Z, Alcaide E, Ke G, Huang H, E W. Accurate Conformation Sampling via Protein Structural Diffusion. *J Chem Inf Model.* 2024;64(22):8414-8426. doi:10.1021/acs.jcim.4c00928
6. Ishima R, Torchia DA, Lynch SM, Gronenborn AM, Louis JM. Solution Structure of the Mature HIV-1 Protease Monomer: insight into the tertiary fold and stability of a precursor. *J Biol Chem.* 2003;278(44):43311-43319. doi:10.1074/jbc.M307549200
